# Calmodulin-like protein AtCML3 mediates dimerization of peroxisomal processing protease AtDEG15 and contributes to normal peroxisome metabolism

**DOI:** 10.1007/s11103-013-0112-6

**Published:** 2013-08-14

**Authors:** Esther Dolze, Fatima Chigri, Timo Höwing, Georg Hierl, Erika Isono, Ute C. Vothknecht, Christine Gietl

**Affiliations:** 1 Institute of Botany, Center of Life and Food Sciences Weihenstephan, TU Munich, Emil-Ramann-Str. 4, 85350 Freising, Germany; 2 Department of Biology, Center of Integrated Protein Science, LMU Munich, 82152 Martinsried, Germany; 3 Department of Plant Systems Biology, Center of Life and Food Sciences Weihenstephan, TU Munich, 85350 Freising, Germany

**Keywords:** AtDEG15, AtCML3, Ca^2+^/calmodulin, Deg/HtrA serine protease, Peroxisomal processing protease

## Abstract

Matrix enzymes are imported into peroxisomes and glyoxysomes, a subclass of peroxisomes involved in lipid mobilization. Two peroxisomal targeting signals (PTS), the C-terminal PTS1 and the N-terminal PTS2, mediate the translocation of proteins into the organelle. PTS2 processing upon import is conserved in higher eukaryotes, and in watermelon the glyoxysomal processing protease (GPP) was shown to catalyse PTS2 processing. GPP and its ortholog, the peroxisomal DEG protease from *Arabidopsis thaliana* (AtDEG15), belong to the Deg/HtrA family of ATP-independent serine proteases with *Escherichia coli* DegP as their prototype. GPP existes in monomeric and dimeric forms. Their equilibrium is shifted towards the monomer upon Ca^2+^-removal and towards the dimer upon Ca^2+^-addition, which is accompanied by a change in substrate specificity from a general protease (monomer) to the specific cleavage of the PTS2 (dimer). We describe the Ca^2+^/calmodulin (CaM) mediated dimerization of AtDEG15. Dimerization is mediated by the CaM-like protein AtCML3 as shown by yeast two and three hybrid analyses. The binding of AtCML3 occurs within the first 25 N-terminal amino acids of AtDEG15, a domain containing a predicted CaM-binding motif. Biochemical analysis of AtDEG15 deletion constructs *in planta* support the requirement of the CaM-binding domain for PTS2 processing. Phylogenetic analyses indicate that the CaM-binding site is conserved in peroxisomal processing proteases of higher plants (dicots, monocots) but not present in orthologs of animals or cellular slime molds. Despite normal PTS2 processing activity, an *atcml3* mutant exhibited reduced 2,4-DB sensitivity, a phenotype previously reported for the *atdeg15* mutant, indicating similarly impaired peroxisome metabolism.

**Electronic supplementary material:**

The online version of this article (doi:10.1007/s11103-013-0112-6) contains supplementary material, which is available to authorized users.

## Introduction

In plants, numerous matrix enzymes have to be imported post-translationally from the cytosol into peroxisomes, including the enzymes for seed storage oil mobilization into glyoxysomes and the components for photorespiration into leaf peroxisomes. The majority of these enzymes are imported in their mature form targeted by a C-terminal (S/A/P/C)-(K/R/S/N)-(L/M/I) tripeptide designated as peroxisomal targeting signal 1 (PTS1) (Lingner et al. 2011). In fewer matrix enzymes, the type 2 peroxisomal targeting signal (PTS2) with the consensus sequence (R/K)-(L/I/V)-X_5_-(H/Q)-(L/A) is located in the N-terminal 30 to 50 amino acids of the pre-protein (Flynn et al. 1998; Reumann 2004; Lazarow 2006). These include glyoxysomal malate dehydrogenase (gMDH), glyoxysomal citrate synthase (gCS), and acyl-CoA oxidase and thiolase in plants, as well as alkyl-dihydroxyacetone-phosphate (DHAP) synthase, phytanoyl-CoA hydroxylase and thiolase in mammals. In higher eukaryotes such as plants and mammals the PTS2 is removed upon import. A Cys residue consistently found in position P2 of the cleavage site (Helm et al. 2007; Schuhmann et al. 2008) is required for efficient processing of the PTS2 pre-sequences of gCS and gMDH in pumpkin (Kato et al. 1996a, 1998). However, processing of PTS2 is not necessary for enzyme activity in higher eukaryotes (Gietl et al. 1996), and it does not take place in lower eukaryotes, where non-cleaved PTS2 sequences are present in the N-terminus of the mature form of thiolase and amine oxidase (Flynn et al. 1998; Helm et al. 2007; Schuhmann et al. 2008).

The purified glyoxysomal processing protease (GPP) from the fat-storing cotyledons of watermelon (*Citrullus vulgaris*) was shown to be responsible for PTS2 processing (Helm et al. 2007). Similarly, lack of the orthologous processing protease AtDEG15 (At1g28320) in an *Arabidopsis* mutant line results in the loss of processing of pre-gMDH to its mature form (Helm et al. 2007; Schuhmann et al. 2008). GPP was shown to exist in two different conformations, a monomer and a dimer. Only in its dimeric form is the protein active as a specific processing protease for PTS2 containing proteins (Helm et al. 2007). In contrast, the monomer was shown to degrade denatured proteins independent from specific sequence motifs. Protein conformation of GPP is calcium-dependent, where the presence of calcium shifts the equilibrium towards the dimeric, PTS2-specific form (Helm et al. 2007) but the molecular basis for this calcium regulation is not known.

DEG15/GPP belong to the Deg/HtrA family of ATP-independent serine endopeptidases with *Escherichia coli* DegP as their prototype (Helm et al. 2007). Members of this family have been shown to form trimeric, hexameric and up to 24-meric complexes (Krojer et al. 2008; Shen et al. 2009). In addition to a domain resembling trypsin, most DegP proteins contain one to three PDZ domains located towards the C-terminus of the polypeptide chain. PDZ domains are about 80 to 90 amino acids long and have been shown to bind to short regions of other proteins (Sheng and Sala 2001). Crystallographic analyses of the trimeric and hexameric forms of *E. coli* DegP suggest that the PDZ domains play a role in substrate recognition and activation of the protease domain (Krojer et al. 2002).

Sixteen genes coding for putative Deg/HtrA proteases have been identified in the *Arabidopsis* genome, six of which are predicted to be targeted to mitochondria and seven to chloroplasts (Huesgen et al. 2005; Schuhmann et al. 2012). AtDEG15 is the only member residing in peroxisomes, as validated by GFP-fusion, proteomics and bioinformatics (Reumann et al. 2004, 2009; Helm et al. 2007; Eubel et al. 2008; Schuhmann et al. 2008).

The Deg/HtrA protease family can be divided into four distinct groups based on differences in their domain structure (Helm et al. 2007; Schuhmann et al. 2012). Group IV comprises AtDEG15 and related enzymes from other organisms such as plant DEG15 orthologs as well as the mammalian peroxisomal processing proteases (HsaTYSND1 and MmuXP125636). Members of this group carry the protease domain more toward the C-proximal part of the protein and they do not contain recognizable PDZ domains. Furthermore, they all possess the capacity to cleave PTS2 sequences. Consequently, DEG15 and TYSND1 were both shown to have a well-defined role in peroxisome biology. The mammalian TYSND1 protease not only removes the targeting signal of PTS2-containing proteins upon import; it also processes certain PTS1-containing peroxisomal fatty acid β-oxidation enzymes (Kurochkin et al. 2007).

Not only DEG15 but also other Deg proteases were shown to alternate between different functional forms, such as the proteolytic and the chaperone function of *E. coli* DegP (Krojer et al. 2008) or the catalytically active and inactive form of *E. coli* DegS (Wilken et al. 2004). While most Deg/HtrA proteases are activated by substrate binding, other mechanisms of activation have been reported including activation by low pH for plant DEG1 (Kley et al. 2011) and bacterial DegQ (Sawa et al. 2011). However, in case of plant DEG15-type proteases, alteration between different forms seems to be Ca^2+^-regulated, and the change between monomer and dimer controls their substrate specificity (Helm et al. 2007). Since DEG15 does not bind the ion itself, Ca^2+^ regulation could be mediated by calcium sensor/transducers such as calmodulin (CaM), a protein found ubiquitously in eukaryotic organisms (Yang and Poovaiah 2003).

CaMs contain two pairs of Ca^2+^ binding EF-hand motifs joined by a linker domain. Upon Ca^2+^ binding, the shape of the CaM molecule changes, triggering its ability to relieve protein autoinhibition, remodel active sites, or dimerize proteins (Clapham 2007). Genomic mining revealed that the *Arabidopsis* genome encodes seven genes for *bona fide* CaMs and about 50 genes for CaM-like proteins (CML) (Day et al. 2002; McCormack et al. 2005). For most CMLs neither function nor localization is known, but AtCML3 (At3g07490) bears the PTS1 motif—SNL at its C-terminus (Reumann et al. 2004). AtCML3 is localized to peroxisomes as validated by targeting of YFP-AtCML3 fusion protein, and it exhibits the typical structure of CaMs (Chigri et al. 2012). AtCML3 furthermore displays common characteristics of canonical CaMs, such as Ca^2+^-dependent alteration of gel mobility and Ca^2+^-dependent exposure of a hydrophobic surface (Chigri et al. 2012). This indicates that AtCML3 can function similar to canonical CaMs and that peroxisomes are integrated into the Ca^2+^/CaM signalling network of the cell.

In this work, we provide experimental evidence for Ca^2+^/CaM-mediated dimerization of AtDEG15. We could further show that AtCML3 interacts with AtDEG15 via a CaM-binding site within the first 25 N-terminal amino acids using yeast two hybrid (Y2H) analyses and bioinformatics. A synthetic peptide corresponding to this domain is able to bind CaM in vitro. CaM-binding at the N-terminus is a pre-requisite for AtDEG15 dimerization as shown by yeast three hybrid (Y3H) experiments. Phylogenetic analyses indicate that the CaM-binding site is conserved in peroxisomal processing proteases of higher plants (dicots and monocots) but not present in orthologs of animals or cellular slime molds. Analysis of an *atcml3* mutant further indicates that loss of AtCML3 impairs peroxisomal metabolism.

## Materials and methods

### Y2H and Y3H

For interaction studies the Matchmaker GAL4 Two Hybrid system (Clontech) was used. Putative interaction partners were cloned into pBridge or pGAD424 vectors in order to achieve fusion proteins with either the DNA-activation domain or the DNA-binding domain. For Y3H, the third protein with its potential regulatory influence was cloned into the second multiple cloning site of pBridge; it is suppressed in the presence of 1 mM Met and is expressed in the absence of Met. For primers see Table S1. All DNA constructs were finally sequenced for control. Plasmids were transformed into *S. cerevisiae* strain AH109; transformands were cultivated according to the manufacturer’s instructions. Two different reporter genes were used: Transcription of the *HIS3* reporter leading to growth on His-free selection medium allows a qualitative estimation of the protein interaction. Transcription of the *lacZ* reporter leads to expression of β-galactosidase converting o-nitrophenol-galactoside to o-nitrophenol; the calculation of Miller units takes into consideration the OD_600_ at the starting point and the reaction time thus allowing a quantitative estimation of the protein interaction dependent on the affinity of the protein partners for each other.

### Growth assay

Transformands were cultivated in 5 ml of YPD medium (1 % yeast extract, 2 % pepton and 2 % glucose). Cell density was defined and concentration was adjusted to 10^5^ cells per 50 μl drop. This suspension was diluted in 1/10 steps until the concentration reached 10^1^ cell per 50 μl drop. Cells were applied on selection medium without supplementation of His to verify His3-reporter gene expression and on selection medium with His as growth control.

### β-Galactosidase assay

Cells were grown for 16 h in selection medium. After incubations, cells were transferred to 4 ml YPD medium and incubated at 30 °C until OD_600_ reached 0.6. Cells from 1 ml were harvested by centrifugation and washed in phosphate buffer pH 7.0 containing 10 mM KCL and 1 mM MgSO_4_. The pellet was resuspended in 100 μl of phosphate buffer, frozen in liquid nitrogen and thawed at 37 °C. This step was repeated twice. 700 μl phosphate buffer pH 7.0 containing 3 mM β-mercaptoethanol was added. Additionally 160 μl of a solution containing 4 mg/ml O-nitrophenyl- β-D-galactopyranoside was added. The solution was incubated at 30 °C for 1 h. After incubation, the reaction was stopped by the addition of 400 μl 1 M Na_2_CO_3_. The solution was centrifuged (Eppendorff centrifuge 5415C, 10 min, 14.000 rpm). Absorption of the supernatant at 420 nm was measured.

$$1\;Miller\;unit = \frac{{\left( {Abs_{420} - \left( {1,75 \cdot Abs_{550} } \right)} \right)}}{{\left( {t \cdot v \cdot Abs_{600} } \right)}}$$

For further details see the manufacturer’s instructions.

### Expression control

To ensure that non-growth observed with certain mutated constructs was not caused by lack of protein expression or protein degradation, yeast proteins were isolated from cells growing on -2 medium (Kushnirov 2000) and analysed by SDS-PAGE. For western blot analysis, antibodies directed against the binding domain in pBRIDGE [GAL4 (DBD) (RK5C1) Santa Cruz Biotechnology] were used.

### Production of recombinant AtDEG15 and AtCML3

For heterologous expression, the coding region of *AtDEG15* or *AtCML3* was cloned into pET28a(+) or pET21a(+) (Merck Chemicals, Darmstadt, Germany; for primers see Tab. S1) to produce recombinant protein with either an N-terminal or C-terminal His-tag and expression was obtained using the Rosetta2 strain (Merck Chemicals, Darmstadt, Germany). AtDEG15 over expressed in *E. coli* yielded the under graded full length AtDEG15 that is, however, deposited in inclusion bodies. From that reason, the purification and subsequent affinity choromatography had to be carried out in the presence of 4 M urea. Cells were harvested 6 h after induction by centrifugation (10 min; 270×*g*), resuspended in 20 mM Tris pH7.5/150 mM NaCl and lysed by sonification. Denatured AtDEG15 was collected by centrifugation (1 h; 75,600×*g*) and solubilized in 4 M urea according to manufacturer’s instructions. Recombinant AtCML3 was soluble and could be purified under native conditions. The proteins were further purified on Ni-TED columns (Machery-Nagel) following the manufacturer’s instructions.

### Production of recombinant AtDEG15_N-terminus

Production of recombinant, soluble AtDEG15 was achieved by using pBAD/His A (Invitrogen, Karlsruhe, Germany): This system produced the AtDEG15 full length protein with an N-terminal His-tag for affinity purification and an N-terminal Xpress™ epitope for immunodetection of the recombinant fusion protein by appropriate antibodies. The protein was produced in low amounts consistently accompanied by AtDEG15 degradation variants (Schuhmann et al. 2008). In analogy to the production of the soluble AtDEG15 full length protein, we cloned the AtDEG15 N-terminus (amino acids 1–327; for primers see Tab. S1), that is the domain N-terminal to the protease domain, into the same vector (pBAD/His A). Expression was induced with 0.02 % l-arabinose. Cells were grown for 16 h at 37 °C after induction, harvested by centrifugation (10 min; 270×*g*), resuspended in 20 mM Tris pH7.5/150 mM NaCl and lysed by sonification. The AtDEG15_N-terminus was expressed as a soluble, undegraded protein and was purified on HisTrap columns under native conditions following the manufacturer’s instructions (GE Healthcare, Freiburg, Germany).

### Affinity chromatography on CaM-agarose

For interaction of AtDEG15 from inclusion bodies with calmodulin, 70 μl CaM-agarose slurry (GE Healthcare, Freiburg, Germany) were washed three times with 300 μl H_2_O and resuspended in 80 μl CaM-binding buffer (20 mM Tris–HCl, pH7.5/150 mM NaCl/0.1 mM CaCl_2_/4 M urea). 20 μg of recombinant, purified AtDEG15 was diluted in CaM-binding buffer (100 μg/ml) and incubated with 40 μl of CaM-agarose-slurry in binding buffer for 16 h at 4 °C. A control experiment with 5 mM EGTA/5 mM EDTA instead CaCl_2_ was performed. The CaM-agarose beads were collected by a brief low-spin centrifugation (1 min/1,000 rpm in an Eppendorff centrifuge). The supernatant (“flow through”) was removed, and the beads were washed 5 times with a tenfold volume of binding buffer with a brief low-spin centrifugation between the washes. For elution of bound proteins, 10 μmol of bovine brain CaM (Calbiochem/Merck, Darmstadt, Germany) or recombinant AtCML3 in 50 μl binding buffer were added three times with a brief low spin centrifugation between the elution steps. The supernatant (“flow through”), the last wash and the three elution fractions, respectively were analyzed by SDS-PAGE and Coomassie staining. For interaction of recombinant, soluble AtDEG15 full length protease and of the AtDEG15 N-terminus, respectively, with calmodulin, the affinity chromatography on CaM-agarose was carried out in a similar manner using native binding buffer (20 mM Tris–HCl, pH7.5/150 mM NaCl/0.1 mM CaCl_2_).

### Cross-linking assays with synthetic AtDEG15 peptide

A synthetic peptide was used comprising the first 21 amino acids of AtDEG15 (H2 N-MDVSKVVSFSRNFAVLVKVEG-CONH2; Eurogentec, Germany). Cross-linking experiments were performed with the zero angstrom cross-linker EDC and sulfo-N-hydroxysulfosuccinimide (Pierce, Germany) as described (Arazi et al. 1995). 300 pmol pig CaM (Enzo Life Sciences, Germany) was incubated in the absence and presence of 600 pmol of synthetic peptide (stock solution 5 mg/ml DMSO) in 50 mM Hepes–KOH (pH 7.5), 150 mM NaCl and 0.1 mM CaCl_2_ for 1 h at 4 °C followed by 30 min cross-linking at RT. Control experiments were performed with 5 mM EGTA and 5 mM EDTA instead of CaCl_2_. As a negative control, the same assays were performed with 300 pmol egg albumin (Sigma, Germany) instead of CaM. The cross-linking reactions were analysed by SDS-PAGE and coomassie staining.

### Analysis of recombinant AtDEG15 enzymatic activity

Cleavage motifs of recombinant, soluble AtDEG15 were analysed by digestion of bovine beta-casein (accession number Swissprot P02666) purchased from Sigma (C-6905). Recombinant AtDEG15 was purified by His-tag affinity chromatography (Ni-TED columns, Machery-Nagel) and incubated with beta-casein (0.13 % final concentration) at 30 °C. Aliquots were taken after 1 min, 4 and 24 h and analysed by 17.5 % SDS-PAGE and coomassie-staining: the signal for undigested beta-casein disappeared, while degradation products increased over time. Digests were stopped by freezing in liquid nitrogen. The same time points were chosen for further analysis: The beta-casein peptides obtained by digestion with AtDEG15 for 1 min, 4 h and 24 h were separated by reversed-phase HPLC prior to MALDI analysis as described (Than et al. 2004). For in vitro transcription/translation the coding sequences of pre-gMDH, pre-gCS and pre-thiolase, respectively, were amplified from RIKEN cDNA clones (pda00743/At5g09660; pda02633/At2g42790; pda07220/At5g48880) using forward primers that included the sequence of the T7 polymerase promotor and a ribosome binding site. Radio-labeled protein was produced from the PCR products using the TNT T7 Coupled Reticulocyte Lysate System from Promega-Deutschland (Mannheim, Germany) for 90 min at 30 °C in the presence of ^35^S-labeled methionine (1,175 Ci/mmol, PerkinElmer LAS Germany GmbH, Rodgau, Germany). The translation mixture was subsequently centrifuged for 20 min at 50,000×*g* to remove aggregated proteins and the supernatant was used in the digestion reaction. 10 μl AtDEG15 (Ni-TED column eluate) were incubated with ten μl substrate (pre-gMDH, pre-gCS or pre-thiolase) in the presence (2 mM Ca^2+^/CaM) or absence (2.5 mM EDTA/EGTA) of CaM at 30 °C for 3 h or over night and analyzed by SDS-PAGE.

### Plant material

*AtDEG15* full-length and deletion constructs were cloned into pET28a(+) (Merck Chemicals, Darmstadt, Germany) followed by cloning into pCAMBIA1301 (Canberra, Australia) under control of the 35S promoter. For PCR primers see Tab. S1. All DNA constructs were finally sequenced for control. *deg15* knock out plants (SALK_007184) were transformed by floral dipping (Prestele et al. 2010). Seeds homozygous for the insertion were germinated, and transcription was assayed in leaves of 20 days old seedlings by RT-PCR using RevertAid First Strand cDNA Synthesis Kit (Fermentas, St. Leon-Rot, Germany). Protein extracts were prepared from 50 pairs of cotyledons grown for 10 days in the dark by Western Blot analysis for gMDH (Gietl et al. 1996) and thiolase (Kato et al. 1996b). Cotyledons were harvested and homogenized in 100 μl grinding medium (125 mM Tris–HCl pH7.5, 1 % SDS, 10 % glycerol and 1 mM DTT). Cell debris was pelleted by centrifugation; the supernatant was analysed on SDS-PAGE.

For 2,4-DB treatment, seeds of Col0 wild type plants and *atdeg15* (SALK_007184) and *atcml3* (pst16586, RIKEN) knock out plants were surface sterilized and sown on agar plates containing 0 μM or 1.8 μM 2,4-DB, respectively, as described (Schuhmann et al. 2008). *atdeg15* and *atcml3* mutant line insertion were sequenced for control.

### Sequence alignment and phylogenetic tree construction

Sequence alignment was obtained by ClustalX 2.0 (Thompson et al. 1997). Phylogenetic tree construction was performed using tree-puzzle-5.2 (Strimmer and von Haeseler 1996). Sequences were obtained from the EMBL/GenBank data libraries. For accession numbers see Table S2A.

## Results

Previous work of our group has shown that the peroxisomal processing protease DEG15/GPP is present in two conformations, monomeric and dimeric, with different substrate specificities (Helm et al. 2007). Homodimerization of DEG15 is calcium-dependent and only the dimeric form of DEG15 represents the PST2-specific peroxisomal protease. So far the molecular basis for the calcium-dependency of dimerization is unknown, and we thus analysed the potential influence of the calcium signal mediator CaM on this process.

### AtDEG15 interacts with AtCML3

We could show recently that the CaM-like protein AtCML3 is localized in peroxisomes (Chigri et al. 2012), making it a potential mediator of calcium-dependent dimerization of AtDEG15. In order to analyse a potential interaction between AtDEG15 and AtCML3, we used the Y2H system with two different reporter genes. Transcription of the *HIS3* reporter leads to growth on His-free selection medium and allows a qualitative or semi-quantitative estimation of protein interaction; transcription of the *lacZ* reporter leads to expression of β-galactosidase and allows a quantitative estimation of protein interaction dependent on the affinity of the protein partners for each other.

**Fig. 1:**
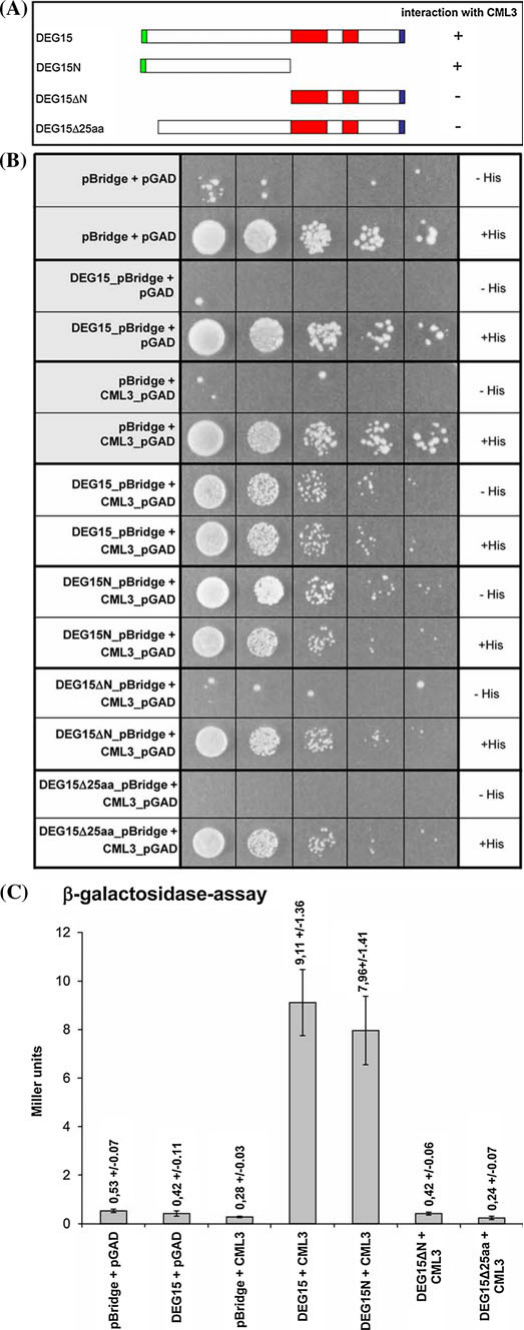
AtDEG15 interacts with AtCML3 through its N-terminal 25 amino acids. To analyse protein interaction by Y2H, the full-length coding regions of AtDEG15, several truncated variants and AtCML3 were cloned into pBridge and pGAD424, fusing them with the DNA-binding domain or with the DNA-activation domain, respectively. As negative controls a combination of empty vectors and vectors harbouring one of the constructs were used. **A** Schematic representation of AtDEG15 and the various deletion constructs used in **B** and **C**: *green*, AtCML3 binding domain; *red*, protease domain with plant-specific loop; blue, PTS1. **B** Growth on His-free medium (−His) indicates interaction by transcription of the His3-reporter; (+His), positive growth control. Negative controls are shaded in grey. Yeast cultures were diluted from 10^5^ cells (*left*) to 10^1^ cells (*right*) per 50 μl drop applied. **C** Interaction is also demonstrated by transcription of the lacZ reporter (β-galactosodase activity in Miller units). Mean (n = 3) ± SD

Initially, interaction of full-length AtDEG15 with AtCML3 was tested together with multiple controls (Fig. 1A and supplementary Fig. S1). The full-length coding regions of *AtDEG15* and *AtCML3* were each cloned both into pBridge and pGAD424, fusing them with the DNA-binding domain or with the DNA-activation domain, respectively. These vectors were then used to double-transform the yeast strain AH109 with either *AtDEG15*_pBridge and *AtCML3*_pGAD424 or vice versa. As negative controls, a combination of empty vectors with complementary vectors harbouring one of the constructs were used. While the results of all experiments are shown in supplementary Figure S1, the most relevant data are presented in Fig. 1. While no growth on His-free plates could be observed for the negative controls, co-transformation of AtDEG15 together with AtCML3—in both combinations—lead to growth of the transformants on His-free medium, indicating that AtDEG15 and AtCML3 interact with each other (Fig. 1B and Fig. S1A). When β-galactosidase activity was analysed, the measured activity for AtDEG15/AtCML3-interaction was 20-fold higher than for the negative controls (Fig. 1C and supplementary Fig. S1B), indicating that the interaction between both proteins is very strong.

The interaction between AtDEG15 and CaM was further confirmed in vitro by affinity chromatography on CaM-agarose (Fig. S2). Using IPTG inducible vectors for production of recombinant AtDEG15 in *E. coli*, expression yielded high amounts of the undegraded AtDEG15 full length protein that, however, was deposited in inclusion bodies. Recombinant 6His-AtDEG15 was isolated from inclusion bodies in the presence of 4 M urea and further purified by His-tag affinity chromatography. Purified AtDEG15 was subsequently incubated with CaM-agarose in the presence of either 0.1 mM CaCl_2_ or 5 mM EGTA/5 mM EDTA and, after extensive washing, the bound proteins were eluted from the matrix by an excess of commercial bovine calmodulin. Protein analysis from all fractions by SDS-PAGE and Coomassie staining revealed binding of AtDEG15 to the CaM-ligand solely in the presence of Ca^2+^ (supplementary Fig. S2A). Furthermore, AtDEG15 could be eluted from the column not only with bovine calmodulin but also with recombinant AtCML3 (supplementary Fig. S2B).

**Fig. 2:**
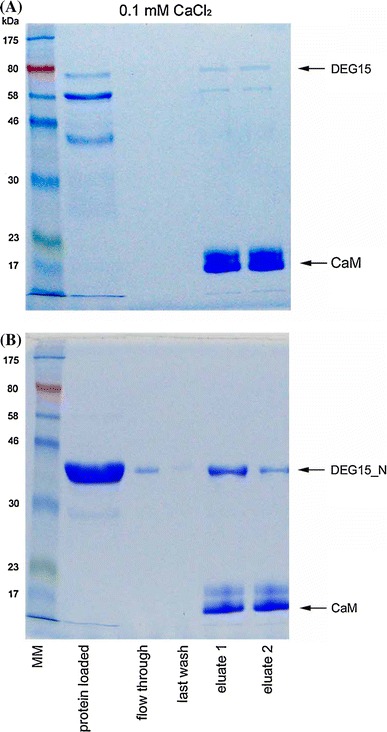
AtDEG15 is a CaM-binding protein as demonstrated by affinity chromatography on CaM-agarose under native conditions. Recombinant full length AtDEG15 (**A**) or a C-terminally truncated protein (AtDEG15_N-terminus; DEG15_N) comprising only the first 327 amino acids without the protease and C-terminal domains (**B**) were incubated with CaM-agarose immediately after His-tag purification in the presence of 0.1 mM CaCl_2_. Bound protein was eluted with an excess of bovine CaM and all fractions were analyzed by SDS-PAGE and Coomassie staining. In case of AtDEG15 several C-terminally truncated degradation products are visible in addition to the full length protein (**A**), the longest of which were also able to bind to the calmodulin-agarose matrix. While AtDEG15 and AtDEG15_N-terminus bound specifically to the matrix in the presence of Ca^2+^ and could be eluted with bovine CaM (**A**) and (**B**), no binding occurred in the absence of Ca^2+^ (5 mM EGTA/EDTA; see supplemental Fig. S2)

We repeated the affinity chromatography on CaM-agarose using recombinant AtDEG15 expressed in *E. coli* under the control of an arabinose-inducible vector (Schuhmann et al. 2008). Production of 6His-AtDEG15 was extremely low, but the protein was produced in a soluble form. However, under these conditions recombinant AtDEG15 protein exhibits the characteristics of the monomer as a general protease with an intrinsic self-cleavage activity. Purification of the N-terminally His-tagged protein consistently resulted in various amounts of degradation products further reducing the amount of full length AtDEG15 (Schuhmann et al. 2008). When His-tag purified AtDEG15 protein was immediately analysed by western blot analysis using antibodies directed against the Xpress-epitope located between the His-tag and the N-terminus of AtDEG15 protein (Fig. S3), the full length AtDEG15 (78 kD including tags) could be detected as well as an ~58 kD protein that still bears the intact N-terminus, since it reacts with the antibodies directed against the Xpress-epitope. This supposingly represents an AtDEG15 variant lacking the 93 amino acids C-terminal to the protease domain, which could be relatively protease-resistant due to its folding (supplementary Fig. S3). When the same purified protein fraction was analysed by affinity chromatography on CaM-agarose (Fig. 2A), the full length AtDEG15 as well as the N-terminally intact AtDEG15 variant bound to the CaM ligand in the presence of Ca^2+^ (Fig. 2A) but not in the presence of EGTA/EDTA (Fig. S2C). Storage of the very same His-tag purified AtDEG15 protein batch over night at 4 °C produced even more N-terminal as well as C-terminal degraded AtDEG15 variants (supplementary Fig. S4A, protein loaded). While all N-terminal intact proteins bound to CaM-agarose, the N-terminal truncated variants did not bind thereby serveing as an internal negative control (Fig. S4A).

Together, these data confirm the specific and Ca^2+^-dependent interaction of AtDEG15 with CaM and suggest an involvement of the N-terminal part of AtDEG15 in this interaction.

### Identification of the AtDEG15 CaM-binding domain

To clearly identify regions important for the interaction with CaM, several N- and C-terminally truncated variants of AtDEG15 were prepared (Fig. 1A; supplementary Fig. S1, inlay) and interaction with AtCML3 was analyzed by Y2H. As already shown above, full-length AtDEG15 interacted with AtCML3, resulting in growth on His-free medium and strong expression of β-galactosidase. Interaction could also be observed with a truncated AtDEG15 variant comprising only the 373 amino acids long N-terminal domain, thus lacking both the protease and the C-terminal domain (AtDEG15N) (Fig. 1B; supplementary Fig. S1A). Quantitative analysis showed that the interaction was strong and at a level similar to the full length AtDEG15 (Fig. 1C; supplementary Fig. S1B). The interaction of AtDEG15_N with CaM in a Ca^2+^-dependent manner was confirmed in vitro by affinity chromatography on CaM-agarose using the AtDEG15_N (aa 1–327) recombinantly expressed in *E. coli* (Fig. 2B; supplementary Fig. S2D).

AtDEG15 variants missing the C-terminal domain or just missing the plant specific loop within the protease domain also interacted with AtCML3 (see Y3H analysis below). In contrast, various N-terminally truncated AtDEG15 constructs did not interact with AtCML3 (Fig. 1B, C; supplementary Fig. S1), confirming that the CaM-binding site is located in the N-terminal part of AtDEG15. Typical CaM binding sites comprise about 25 amino acids (Calmodulin Target Database CTDB: http://calcium.uhnres.utoronto.ca/). We thus narrowed down the CaM-binding domain by deleting the first 25, 50, 75 or 100 amino acids of AtDEG15, respectively (Fig. 1 and Fig. S1). Indeed, already the removal of the first 25 amino acids was sufficient to completely abolish interaction of AtDEG15 with AtCML3 suggesting that the CaM-binding site is located in the outmost N-terminus of AtDEG15 (Fig. 1B, C; Fig. S1).

To ensure that the results observed with N-terminally truncated variants are not caused by lack of expression or protein degradation, we analysed the presence of these variants by western blot. Western blot analysis of the most relevant constructs using antibodies directed against the BD in pBridge confirmed that the AtDEG15 recombinant proteins are indeed being expressed in yeast and are of the expected size (supplementary Fig. S5).

**Fig. 3:**
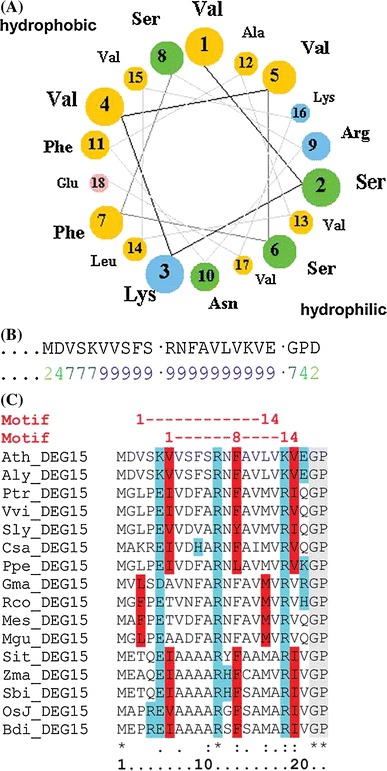
Bioinformatic analyses of the putative CML-binding domain in the N-terminal 20 amino acids of plant DEG15. **A** Helical wheel projection of AtDEG15 Val-3 to Glu-20 reveals a basic amphipathic helix structure (http://cti.itc.virginia.edu/~cmg/Demo/wheel/wheelApp.html). *Yellow*, nonpolar aa; *green*, polar, uncharged aa; *red*, acidic aa; *blue*, basic aa. **B** *Calmodulin target database* (CTDB, http://calcium.uhnres.utoronto.ca/) calculates the highest possible probability (0–9, with 9 being the highest probability) for a CaM binding motif between AtDEG15 Val-6 and Glu-20. **C** Two established Ca^2+^-dependent CaM-binding motifs, 1–8–14 and 1–14 based on the spacing between the hydrophobic residues (*red*), are found within the N-terminal 23 amino acids; charged residues in the amphipathic helix are colored *blue*; the conserved GP-linker C-terminal to the CaM-binding motif is *shaded in grey*. The motifs are conserved between dicots and monocots. Ath, *Arabidopsis thaliana*; Aly, *Arabidopsis lyrata*; Ptr, *Populus trichocarpa*; Vvi, *Vitis vinifera*; Sly, *Solanum lycopersicum*; Csa, *Cucumis sativus*; Ppe, *Prunus persicus*; Gma, *Glycine max*; Rco, *Ricinus communis*; Mes, *Manihot esculenta*; Mgu, *Mimulus guttatus*; Sit, *Setaria italica*; Zma, *Zea mays*; Sbi, *Sorghum bicolour*; OsJ, *Oryza sativa* Japonica; Bdi, *Brachypodium* *distachyon*. For accession numbers see Supplemental Tab. S2A

Bioinformatic analyses supported the presence of a putative CaM-binding site at the very N-terminus of AtDEG15 (Fig. 3). A characteristic of CaM-binding domains is the spacing between two bulky hydrophobic residues and the formation of a basic amphiphilic helical structure and the latter is formed between Val-3 and Glu-20 of AtDEG15 (Fig. 3A). A high probability for a CaM-binding motif is indicated within the same first 20 N-terminal amino acids of AtDEG15 (Fig. 3B; Calmodulin Target Database, CTDB), as well as both a so-called 1–8–14 and a 1–14 motif (Fig. 3C). To confirm these bioinformatic data, we performed crosslinking experiments with CaM and a synthetic peptide comprising the first 21 amino acids of AtDEG15 (H2N-MDVSKVVSFSRNFAVLVKVEG-CONH2). A cross-linking product with CaM can be observed solely in the presence of both the synthetic peptide and calcium (supplemental Fig. S6A; +calcium). No cross-linking product occurs in the absence of calcium (Fig. S6A; −calcium) or when CaM is replaced by egg albumin (Fig. S6B). This supports that the first 21 amino acids of AtDEG15 are able to specifically interact with CaM.

A sequence alignment of various plant DEG15 orthologous revealed highly conserved 1–8–14 or 1–14 motifs within the N-terminal 20 amino acids of all proteins from dicots and monocots (Fig. 3C; supplemental Fig. S7). By contrast, the moss sequence (PhpaDEG15) has no clear CaM binding motif at the beginning of its long N-terminus (Fig. S7). The alignment shows that PhpaDEG15 contains the conserved methionine that is positioned at the start of the CaM binding motif in higher plants, whereas several of the hydrophobic and basic amino acids that are necessary and conserved in higher plants for formation of the amphiphilic helix are missing within this stretch of amino acids (Fig. S7). As a consequence, the prediction programs do not indicate a CaM binding motif within the moss sequence.

Together these results suggest that the first 20–25 amino acids of AtDEG15 are necessary and responsible for CaM-binding. No CaM-binding motif is found within the N-terminus of peroxisomal processing proteases from animals and cellular slime molds or from the moss *Physcomitrella patens* (Fig. S7), indicating that CaM-binding together with Ca^2+^-dependent regulation of DEG15 dimerization is conserved among and specific for higher plants (monocots and dicots).

### Homodimerization of AtDEG15 requires calmodulin

**Fig. 4:**
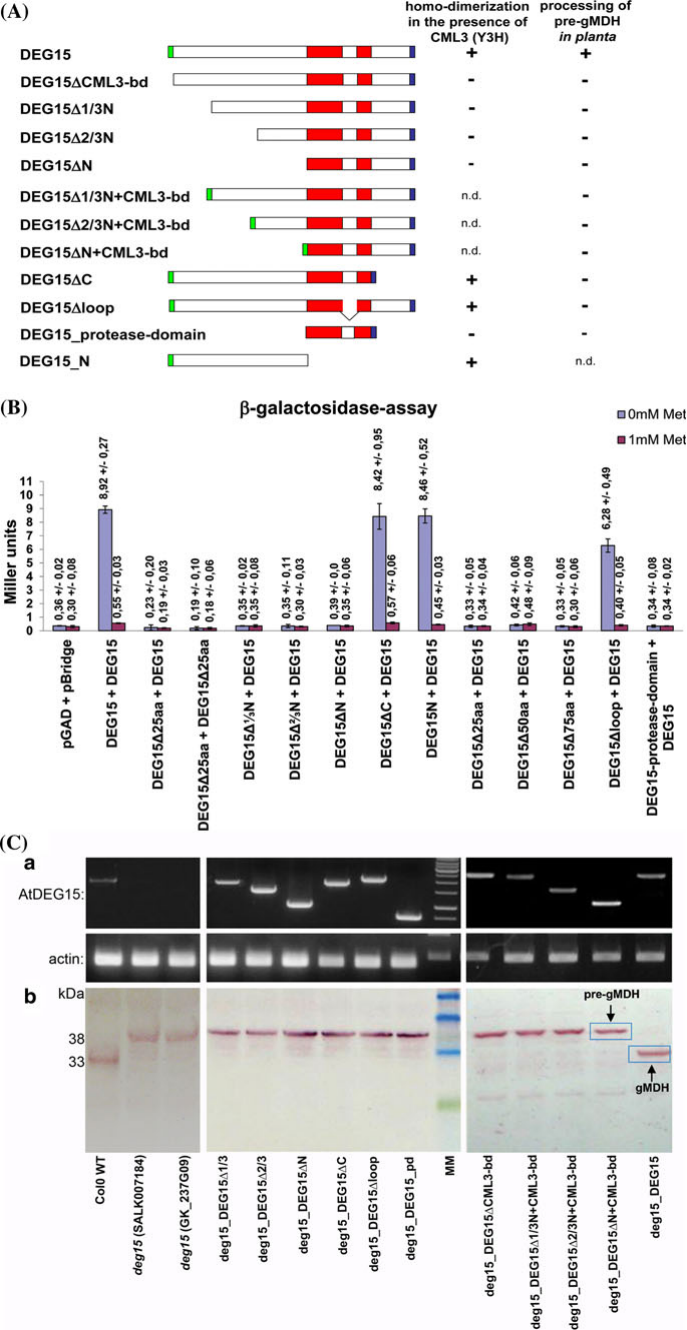
AtDEG15 homodimerizes in the presence of AtCML3 as shown by Y3H. Only the full-length AtDEG15 processes PTS2 *in planta*. **A** Schematic representation of AtDEG15 and the various deletion constructs analyzed by Y3H and/or by transformation into the *A. thaliana* *deg15* insertion mutant plant SALK007184; green, AtCML3 binding domain; red, protease domain with the plant-specific loop; blue, PTS1; nd, not determined. **B** Interaction assay using Y3H. The full length AtDEG15 was cloned in pBridge; the various AtDEG15 deletion constructs were cloned in pGAD424. AtCML3 was cloned into the second multiple cloning site of pBridge; it is expressed in the absence of methionine (0 mM Met) and is repressed in the presence of methionine (1 mM Met). Interaction of AtDEG15 with AtDEG15 is strong in the presence of AtCML3; no homodimerization takes place in the absence of AtCML3. Both AtDEG15 molecules need the CML binding domain to achieve homodimerization. The domain N-terminal to the protease domain, especially the CML binding domain in the 25 N-terminal amino acids is necessary for homodimerization. Mean (n = 3) ± SD. **C** Analysis of PTS2 processing ability of AtDEG15 and its deletion constructs *in planta* after transformation into the *deg15* KO mutant plant (SALK007184). Left: PTS2 processing ability of WT Col0 and *deg15* mutant lines SALK007184 and GABI-Kat GK_237G09; middle and right: mutant line SALK007184 transformed with the various AtDEG15c constructs. (*a*) The transformed constructs are transcribed as shown by RT-PCR. (*b*) Only full-length AtDEG15 processes pre-gMDH to the mature subunit as shown by westernblot analysis with α-gMDH antibodies. Loss of N-terminal domains, loss of the C-terminus or of the loop within the protease domain abolishes processing. Deletion of N-terminal domains despite including the AtCML3-binding sequence also abolishes PTS2 processing. MM, DNA marker (GeneRuler 1 kb DNA Ladder, Fermentas) and prestained protein molecular weight markers (blue: 58 kDa, 46 kDa, 30 kDa; green 23 kDa; BioLabs), respectively

Y2H analysis (Fig. 1 and Fig. S1), affinity chromatography on CaM-agarose (Fig. 2 and Fig. S2), bioinformatic analyses (Fig. 3) and CaM-AtDEG15 peptide interaction (Fig. S6) all support an interaction between AtDEG15 and AtCML3 due to CaM-binding at the most N-proximal 20 amino acids of AtDEG15. Since it was previously shown that homodimerization of DEG15/GPP is calcium-dependent (Helm et al. 2007), a potential role of AtCML3 in this process was elucidated by Y3H analysis using several deletion variants of AtDEG15 (Fig. 4A) in the presence or absence of AtCML3.

Homodimerization of full length AtDEG15 was strong in the presence of AtCML3, reaching 8.92 Miller units (Fig. 4B; DEG15/DEG15, 0 mM Met). By contrast, homodimerization of full length AtDEG15 in the absence of AtCML3 was at the level of the empty vector control. Interestingly, both AtDEG15 molecules need to contain the CaM-binding domain, since no effect of AtCML3 on homodimerization could be observed, if a full length AtDEG15 was tested against the construct AtDEG15ΔCML3-bd lacking the N-terminal 25 amino acids long CaM binding domain (Fig. 4B; DEG15Δ25aa/DEG15). Consequently, no homodimerization in the presence of AtCML3 was observed either, when both AtDEG15 molecules lacked this domain (DEG15Δ25aa/DEG15Δ25aa) or in all other constructs lacking this part of the protein (Fig. 4B). By contrast, the N-terminus of AtDEG15 always promoted homodimerization in the presence of AtCML3 (Fig. 4B; DEG15Δloop/DEG15; DEG15ΔC/DEG15), even if present alone (Fig. 4B; DEG15N/DEG15).

These findings support the conclusion that CaM binding to AtDEG15 at the N-terminal CaM-binding domain is a decisive feature for homodimerization of AtDEG15 and that AtCML3 is a potential mediator of this process.

### Full length AtDEG15 is required for restoration of PTS2 processing in *atdeg15* mutant *in planta*

**Fig. 5:**
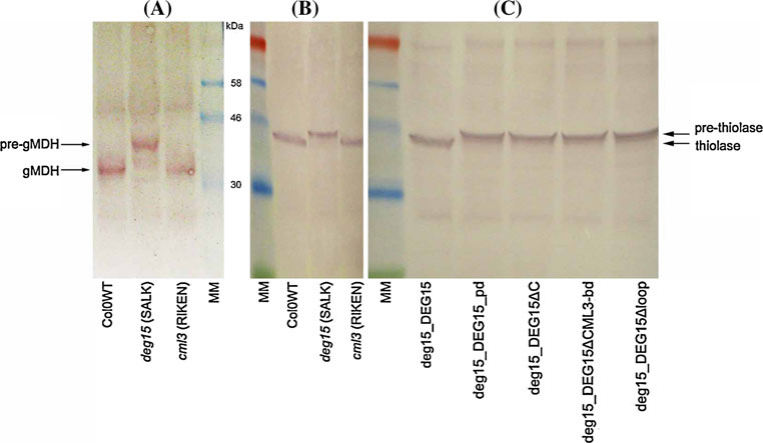
Analysis of PTS2 processing ability of *atcml3* and *atdeg15* mutant lines. Only the full-length AtDEG15 processes pre-thiolase to the mature thiolase *in planta*. **A** and **B** The *atcml3* mutant line (pst16586, RIKEN)—unlike the *atdeg15* mutant line (SALK007184)—is not impaired in processing of pre-gMDH to the mature gMDH (**A**) or pre-thiolase to the mature thiolase (**B**) as shown by western blot analysis of protein extracts using antibodies directed against gMDH or thiolase. **C** Analysis of several AtDEG15 variants shows that only full-length AtDEG15 processes pre-thiolase to mature thiolase as shown by western blot analysis with α-thiolase antibodies. Loss of the N-terminal CML binding-domain, loss of the C-terminus, loss of N- and C-terminus or of the loop within the protease domain all abolish processing. MM, prestained protein molecular weight markers (*red*, 80 kDa; *blue*: 58, 46, 30 kDa; *green* 23 kDa; BioLabs)

As shown above, CaM-binding at the N-terminus of AtDEG15 is a pre-requisite for homodimerization. This is important since only the DEG15 dimer is a specific PTS2 processing protease (Helm et al. 2007). We thus transformed AtDEG15 variants corresponding to those used in the Y3H experiments (Fig. 4A) into *atdeg15* knockout mutant plants of *A. thaliana* (SALK_007184) under control of the 35S promoter. All AtDEG15 variants had the C-terminal PTS1–SKL attached in order to ensure their correct targeting into peroxisomes, and transcription of all constructs was confirmed by RT-PCR (Fig. 4C a). Successfully transformed *atdeg15* knockout plants homozygous for the insertion of the different AtDEG15 variants were then used to analyze processing of the PTS2-containing enzymes pre-gMDH and pre-thiolase *in planta* (Figs. 4C, 5).

Processing of pre-gMDH to the smaller mature gMDH is easily visible by Western Blot analysis probed with a gMDH-specific antiserum when wild type protein extracts (Fig. 4C b; Col0 WT) are compared to knockout mutants of *atdeg15* (Fig. 4C b; compare WT, SALK_007184 and GABI-Kat line GK_237G09). Transformation of the SALK_007184 mutant plant with a full-length AtDEG15 construct restored the processing of pre-gMDH to mature gMDH (Fig. 4C b; deg15_DEG15). Obviously the full length AtDEG15 is present and functional in the transformed plants as can be recognized by its processing activity, thus confirming the feasibility of this experimental approach. Transformation of the SALK_007184 mutant plant with diverse AtDEG15 variants lacking the calmodulin-binding domain or with the DEG15 protease domain alone could not restore the pre-gMDH to gMDH processing (Fig. 4C; deg15_DEG15ΔCML3-bd, deg15_DEG15Δ1/3, deg15_DEG15Δ2/3, deg15_DEG15ΔN, deg15_DEG15Δpd).

AtDEG15 proteins missing the domain C-terminal to the protease domain or missing the plant-specific loop within the protease domain also did not process pre-gMDH (Fig. 4C; deg15_DEG15ΔC, deg15_DEG15Δloop) although they contain the calmodulin-binding domain and were able to homodimerize as shown by Y3H analysis (Fig. 4B). Furthermore, AtDEG15 constructs lacking different amounts of the N-terminal domain but containing the calmodulin-binding domain artificially attached at the N-terminus were also not able to process pre-gMDH *in planta* (Fig. 4C; deg15_DEG15Δ1/3Ν + CML3-bd, deg15_DEG15Δ2/3Ν + CML3-bd, deg15_DEG15ΔΝ + CML3-bd). This shows that multiple domains of AtDEG15 are required for proper processing enzyme activity. The AtDEG15-N-terminus alone was not transformed into the *atdeg15* knockout mutant (SALK_007184), since processing activity is not to be expected without the protease domain.

Processing of pre-thiolase to the smaller mature thiolase is also easily visible by Western Blot analysis probed with a thiolase-specific antiserum. The most relevant protein extracts used above were thus also analyzed for pre-thiolase processing ability (Fig. 5). Again, the *deg15* knockout mutant plant could not process pre-thiolase (Fig. 5B). Transformation of the *deg15* knockout mutant plant with the full length AtDEG15 construct restored processing, whereas the various deletion constructs did not restore processing enzyme activity (Fig. 5C).

### Recombinant AtDEG15 exhibits the characteristics of the monomer as a general protease with an intrinsic self-cleavage activity

We tried to confirm the necessity of CaM for AtDEG15 PTS2 processing activity in vitro using the recombinant, enzymatically active AtDEG15. Unfortunately, the enzyme exhibited only the general protease activity described for the monomeric form. Digestion of bovine β-casein with recombinant AtDEG15 revealed several peptides as identified by MALDI-TOF (supplemental Fig. S8), deriving especially from cleavage within the C-terminal part of β-casein. When the recombinant AtDEG15 was incubated with ^35^S-methionine labeled pre-gMDH, pre-gCS or pre-thiolase, it processed pre-gMDH to mature gMDH, albeit independent of the presence or absence of calcium, while neither pre-gCS nor pre-thiolase were processed under either condition. It is important to notice, that pre-gMDH is not an indicative substrate in vitro since processing at the correct cleavage site can be achieved also by other unrelated proteases such as the ricinosome-localized KDEL-tailed cysteine endopeptidase involved in programmed cell death (Gietl et al. 1997; Schmid et al. 1998). This is probably due to an unusual distribution of amino acids at the cleavage site (Helm et al. 2007).

As mentioned above, the recombinant AtDEG15 also seems to digest itself leading to multiple N-and C-terminal truncated variants of the protein (Figs. 2A, S3 and S4A). By contrast, the AtDEG15 N-terminus lacking the protease domain and C-terminus was stable for a much longer time and also interacted with the CaM-ligand (Figs. 2B and S4B). Unfortunately, it was not possible to isolate the AtDEG15 full length protein and remove all cleavage products by size exclusion chromatography since storage or further analysis of the isolated AtDEG15 full length protein resulted again in degradation variants.

Since the monomeric form of AtDEG15 is a general protease activated by denatured proteins (Helm et al. 2007), we would thus assume, that correct folding of the entire recombinant AtDEG15, especially of the long N-terminus as a pre-requisite for dimeric PTS2-specific enzymatic activity cannot be ensured in vitro, thereby resulting in protein auto-degradation.

### Role of CaM/AtCML3 in homodimerization of AtDEG15 and in peroxisome metabolism

The data presented so far indicate that CaM binding promotes homodimerization of AtDEG15, which in turn is a necessary pre-requisite for its PTS2 processing activity. While AtCML3 can mediate AtDEG15 homodimerization, it is unclear whether AtCML3 or a different CML full-fills this role *in planta*. The *atcml3* knockout mutant line (pst16586, RIKEN) has a transposon insertion directly in front of the PTS1 signal—SNL of AtCML3. This would prevent peroxisomal targeting presumably leading to mislocalization of AtCML3 to the cytosol as shown in our previous publication for a YFP-AtCML3 fusion construct lacking SNL (Chigri et al. 2012). Nevertheless, PTS2 processing of pre-gMDH to the smaller mature gMDH and of pre-thiolase to the smaller mature thiolase, respectively, is unimpaired in the *atcml3* knockout mutant line as shown by Western Blot analysis of protein extracts probed with the gMDH- and the thiolase-specific antiserum, respectively (Fig. 5A, B).

**Fig. 6:**
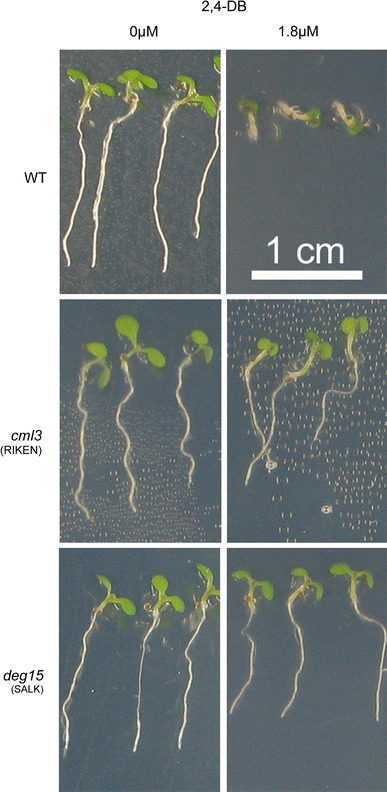
Resistance of *atcml3* and *atdeg15* knock out plants to the herbicide precursor 2,4-DB. Photographs show seedlings grown for seven days on solid medium containing 0 μM or 1.8 μM 2,4-DB. WT, wild type plants; *cml3*(RIKEN), *atcml3* knock out plants pst16586; *deg15*(SALK), *atdeg15* knock out plants SALK007184

However, *atcml3* knockout plants do show defects in normal peroxisomal metabolism. Treatment of wild type plants with the herbicide precursor 4-(2,4-dichlorphenoxy) butyric acid (2,4-DB), which is converted in peroxisomes to the toxic herbicide 2,4-dichlorphenoxyacetic acid (2,4-D) by beta-oxidation, results in a strong reduction of root growth. A resistance to 2,4-DB can also be observed for homozygous *atcml3* mutants when grown on solid medium containing 2,4-DB (Fig. 6). Interestingly, it was shown recently that the lack of PTS2 processing by AtDEG15 results in an increased resistance to the 2,4-DB, the only obvious phenotype observed so far for homozygous *atdeg15* mutant plants (Schuhmann et al. 2008).

Thus, not only seems AtCML3 to be involved somehow in peroxisomal β-oxidation; the phenotypical similarity between for *atcml3* and *atdeg15* mutants corroborates a functional correlation between these two proteins in vivo.

## Discussion

In this study we analyzed the Ca^2+^/CaM dependent homodimerization of the plant peroxisomal processing protease AtDEG15. A specific feature of AtDEG15 and its higher plant orthologues is that a change in substrate specificity is coupled to a change in their oligomeric states. The protein acts in the monomeric conformation as a general protease cleaving at Φ-Arg/Lys↓-, while the dimeric form cleaves the PTS2-containing N-terminal presequence of higher eukaryotes at Cys-Xxx↓/Xxx-Cys↓-during peroxisomal protein import. It has been shown previously that the dimerization of DEG15 proteases requires calcium but the mode of calcium action remained unknown (Helm et al. 2007).

Interaction between AtDEG15 and peroxisomal AtCML3 was demonstrated by Y2H and Y3H analysis and by affinity chromatography on CaM agarose, which also allowed the identification of the putative CaM-binding side within the first 25 N-terminal amino acids of AtDEG15. This part of AtDEG15 exhibits a classical CaM-binding motif (*Calmodulin target database*) and the motif is highly conserved among plant DEG15 proteins. Crosslinking experiments with CaM and a synthetic peptide comprising the first 21 amino acids of AtDEG15 confirmed this interaction. Together, these data suggest that this N-terminal CaM-binding motif is responsible for the interaction between plant DEG15 and CaM.

Unfortunately, it is nearly impossible to confirm dimer-specific processing activity in vitro. Recombinant AtDEG15 is enzymatically active in vitro as a general protease since it can digest milk beta-casein. This is consistent with the presence of recombinant AtDEG15 in its monomeric form. Recombinant AtDEG15, however, does not process pre-gCS or pre-thiolase. This might be due to only partial folding of recombinant AtDEG15. In contrast to the central protease domain, both the N-terminal and the C-terminal domain are characterized by an unusual high portion of Gly and Pro (see colouring in Fig. S7), which might give a flexible structure to AtDEG15 and thus abolish correct folding in vitro. As a consequence, the N-terminus, which is decisive for the Ca^2+^/CaM-dependent homodimerization of AtDEG15, might not exhibit the appropriate folding as it is achieved *in planta*. The DEG15 N-terminus is unique with no homologue domains/proteins found in the databases including DEG-proteases from animals and yeast databases. Its concrete function is unknown and will be subject to further analysis. By contrast to the in vitro data, the full length *AtDEG15* transformed into the *atdeg15* mutant line could restore the PTS2 processing activity *in planta*. None of the various *AtDEG15* deletion variants was able to do so, suggesting that CaM-induced homodimerization is a pre-requisite for PTS2 processing in vivo. While the mRNA of *AtDEG15* and its variants was detectable. The proteins were below the detection level of our antibody and it can thus not fully be excluded that PTS2 processing is not restored due to a lack of active protein. However, we could demonstrate in vivo by Y2H and Y3H analysis that only full length AtDEG15 protein can homodimerize through CaM-mediation, whereas the AtDEG15 protein lacking just the N-terminal 25 amino acids failed to interact with AtCML3 and consequently shows no CaM-mediated homodimerization.

A role of AtCML3 in peroxisomal metabolism was confirmed by the resistance of the *atcml3* knockout mutant to the inhibitory effects of 2,4-DB in root elongation. The IBA analog 2,4-DB is thought to be converted to the synthetic auxin 2,4-D in a pathway similar to the IBA-to-IAA conversion. The conversion is similar to the peroxisomal fatty acid β-oxidation (Zolman et al. 2007). By analogy to fatty acid and IBA β-oxidation, several enzymes should be involved in 2,4-DB conversion to 2,4-D. One of these enzymes is likely to be the PTS2-containing 3-ketoacyl-CoA thiolase (At2g233150) encoded by *PED1* also known as KAT2 as *ped1* null mutants are 2,4-DB resistant (Hayashi et al. 1998; Germain et al. 2001; Burkhart et al. 2013). Furthermore, PED1 was found to be important for production of reactive oxygen species (ROS) in response to ABA (Jiang et al. 2011). IBA-response mutants fall into two categories: those with general peroxisomal defects, including reduced rates of fatty acid β-oxidation, and those that are IBA-resistant but appear to carry out other peroxisomal functions normally (Zolman et al. 2008). *ped1*/*kat2* belongs to the first category. The latter category includes mutants of the loci *ibr3*, *ibr10* and *ibr1* encoding three novel peroxisomal enzymes: a novel acyl-CoA dehydrogenase-like protein (IBR3), an enoyl-CoA dehydrogenase like protein (IBR10) and a member of the short-chain dehydrogenase/reductase (IBR1), which may act sequentially in peroxisomal β-oxidation of IBA to IAA (Zolman et al. 2007, 2008). All three proteins contain C-terminal peroxisomal targeting signals and are not subject to PTS2 processing. The *atdeg15* and the *atcml3* knock out mutant lines fall in the same category since they have no obvious defect in germination and storage oil mobilization but are resistant to the inhibitory effects of 2,4-DB.

Based on bioinformatics and biochemical analyses, AtCML3 seems to be the only CaM-like protein localized within peroxisomes since no other CaM or CML in *Arabidopsis* contains a recognizable peroxisomal targeting sequence (Reumann et al. 2004; Chigri et al. 2012). It is thus surprising that an analysis of an *atcml3* mutant line, expressing AtCML3 without its PTS1 targeting sequence revealed unimpaired pre-gMDH and pre-thiolase processing suggesting that AtCML3 contributes to normal peroxisome metabolism but might not be involved in PTS2 processing. However, it is well established that the peroxisomal protein import machinery allows for the translocation of fully folded, co-factor bound and even oligomeric proteins across the peroxisomal membrane; proteins that lack a PTS can be sorted to peroxisomes when in complex with a PTS-containing subunit (Brown and Baker 2008; Girzalsky et al. 2010). We did not observe such co-import of YFP-AtCML3 lacking its PTS1 into peroxisomes in our previous publication (Chigri et al. 2012) but this could have been due to the expression of YFP-AtCML3 lacking its PTS1 in the wild type background with the endogenous AtCML3 still present. As a consequence, the plant has “no reason” for a “piggy-back” import of a C-terminally truncated AtCML3 even loaded with the YFP-fusion. In addition, the strong presence of the cytosolic C-terminally truncated YFP-AtCML3 might cover small amounts of co-imported YFP-AtCML3 in the peroxisome. Furthermore, plants possess a large number of CaM and CML proteins (e.g. more than 50 in *Arabidopsis*). For most CMLs, their specific target proteins are unknown and—at least in vitro—are rather indiscriminating, as evidenced by the common use of bovine brain CaM as the ligand for column purification of CaM-binding proteins. It cannot be excluded that AtDEG15 interacts with yet another CaM/CML followed by co-import into peroxisomes, either specifically or as a way for the plant to overcome the lack of peroxisomal AtCML3. Also. another so far undetected peroxisomal CML cannot be excluded since several *CML* genes seem to be prone to alternatibve splicing. A general lack of any outward phenotypic effect had been observed before for the *atcml3* mutant (Hanada et al. 2009), and the authors suggested the phenomenon of functional compensation of (duplicate) genes within the CaM/CML gene family in *Arabidopsis* as a possible explanation.

So while the 2,4-DB resistance of *atdeg15* and *atcml3* indicates that these two proteins likely have a correlating function in vivo, their specific role in peroxisome metabolism needs further investigation.

Enzyme regulation by Ca^2+^/CaM is rather common in plants and the number of CaM-target proteins identified is increasing (Yang and Poovaiah 2003; Popescu et al. 2007). More than 20 plant-specific CaM-binding proteins with no obvious homologs in other organisms have been identified, and about 20 CaM-targets have homologs in other organisms but are probably regulated by CaM only in plants (for review see Bouche et al. 2005). A recent example is the *Arabidopsis* AAA^+^-ATPase AFG1L1 (At4g30490), which seems to bind CaM in a Ca^2+^-dependent fashion within its catalytic AAA-domain in plants but not in animals and fungi (Bussemer et al. 2009). Another intriguing example of enzymes that have acquired CaM-regulation during evolution are kinesins, a superfamily of microtubule (MT) motors. Plants are unique in containing a large number of kinesins (61 in *Arabidopsis*) including several that are plant specific (Bouche et al. 2005). One of the plant kinesins, kinesin-like CaM-binding protein (KCBP) was isolated from dicot and monocot plants as a CaM-interacting protein (Reddy et al. 1996; Wang et al. 1996) but no CaM-binding kinesin has been identified in fungi, *C. elegans*, *D. melanogaster*, or *H. sapiens*. KCBP is involved in plant specific processes such as trichome morphogenesis and is associated with preprophase band and phragmoplast in dividing cells, which are absent in animal cells. CaM inhibits the motor activity of KCBP and its interaction with MTs in a Ca^2+^-dependent manner. By fusing the CaM-binding domain of KCBP to a *Drosophila* kinesin it could be shown that this domain can function as a regulatory module in canonically non-CaM-binding kinesins (Reddy and Reddy 2002). The plant peroxisomal processing protease DEG15 seems to be a similar example for an acquired CaM regulation during evolution. Further studies, especially co-crystallization of the dimeric DEG15 complex with CaM should elucidate how this interaction modulates structure and substrate specificity.

It is also noteworthy, that the calcium-mediated dimerization of AtDEG15 and its contribution to normal peroxisome metabolism is not the only peroxisomal process supposedly regulated by CaM/CML. Plant catalase, which scavenges H_2_O_2_ generated during β-oxidation of fatty acids and photorespiratory oxidation in peroxisomes, was shown to be activated by Ca^2+^/CaM (Yang and Poovaiah 2002). *Arabidopsis* peroxisomes were also shown to undergo Ca^2+^ fluxes, and intra-peroxisomal Ca^2+^ rise in vivo stimulates catalase activity thus increasing peroxisomal H_2_O_2_ scavenging efficiency (Costa et al. 2010). Peroxisomes participate in the Ca^2+^/CaM signaling pathway of the cell (reviewed in Stael et al. 2012). Differential regulation of various peroxisomal enzymes by Ca^2+^/CaM might thus require the use of different CMLs.

CaMs and the plant specific CMLs are among the most prominent Ca^2+^ transducers in eukaryotic cells, regulating the activity of numerous proteins with diverse cellular functions. Several protein activation/inhibition mechanisms have emerged, including relief of auto-inhibition, active site remodeling, dimerization, stabilization of multimeric complexes and inactivation by occupying a ligand-binding site (Yang and Poovaiah 2003; Bouche et al. 2005). Regulation of proteases through CaM is not widespread, but there are other examples such as the Ca^2+^-dependent cysteine protease calpain (EC3.4.22.17) and its endogenous inhibitor protein calpastatin in animals. They interact through CaM-like domains in the calpain large and small subunits in a Ca^2+^-dependent fashion and kinetic analyses of the calpain-calpastatin interactions indicate a competitive type inhibition (Kawasaki et al. 1989; Takano et al. 1995). We could demonstrate that the CaM-binding site at the N-terminus of each AtDEG15 molecule is required for CaM-mediated dimerization and a single CaM-binding to both sites could be responsible for connecting two AtDEG15 molecules. It is also possible that one end of the CaM binds to the N-terminus whereas the other end interacts with a potential second CaM-binding motif within the protease domain of AtDEG15. This mechanism would resemble the dimerization of CaM shown for the small conductance Ca^2+^-activated potassium channel by the 3D structure. In this case, Ca^2+^ ions are bound only to the N-terminal EF-hands while the C-terminal EF motifs mediate tethering to the channel (Bouche et al. 2005).

**Fig. 7:**
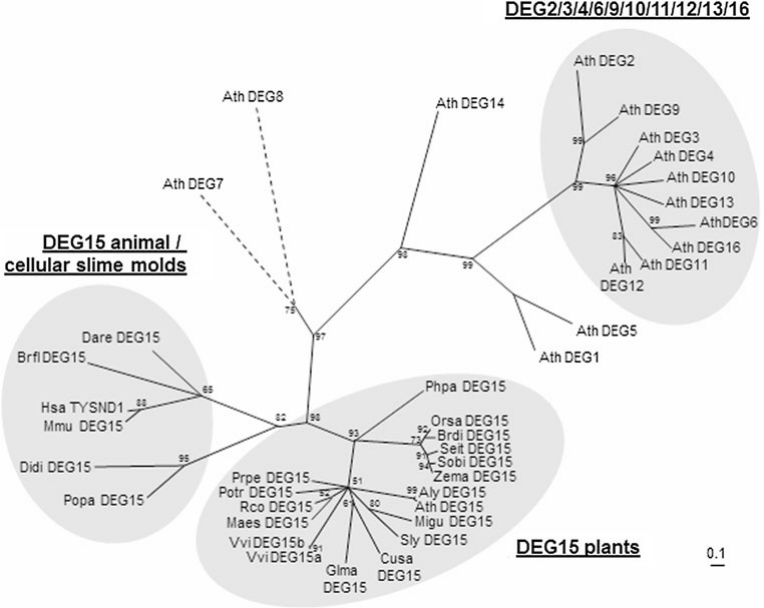
Phylogenetic relationship of peroxisomal processing proteases from plants, animals and cellular slime molds and of DEG proteases in *Arabidopsis*. The *dotted lines* indicate that this part of the tree is not drawn to scale. Sequence alignemnts were obtained by ClustalX 2.0 (Thompson et al. 1997) and phylogenetic tree construction was performed by maximum likelihood using tree-puzzle-5.2 (Strimmer and Haeseler 1996). The alignment on which the phylogenetic tree was based is provided in Supplemental Fig. 7. For accession numbers see Supplemental Tab. S2A

Plant DEG15/GPP proteases exhibit several unique features. BLAST searches identified DEG15 orthologs in at least 10 dicots, 5 monocots and the moss *Physcomitrella patens* (Supplementary Fig. S7; supplementary Tables S2A and S2B). While all other sequences are similar in length, the moss sequence is significantly prolongated due to a long N-terminal extension (Fig. S7, Table S2A). DEG15-like proteases are also found in *Medicago trunculata*, *Aquilegia coerulea* and *Carica papaya*, but the sequences available in the databases appear to be truncated and were thus not considered for comparisons (Table S2B). Proteases with a PTS1 were also found in the green algae *Volvox carteri f. nagariensis* and *Micromonas sp.* RCC299; however, the catalytic His is missing and they might thus belong to a different class of peroxisomal proteases (Table S2B). Most plants contain a single *DEG15* gene, but some plants such as *V. vinifera* (Tab. S2A) or *P. trichocarpa* (supplementary Tables S2A and S2B) have more than one DEG15 protease differing by the presence of peptide insertions in non-conserved domains. DEG15 orthologs can further be found in animals and cellular slime molds (Figs. S7 and S9; Table S2A). Sequence comparison revealed that all DEG15-like peroxisomal processing proteases are characterized by a C-terminal non-cleavable PTS1 and the catalytic triade His-Asp-Ser. By contrast, the loop within the protease domain between the catalytic His and Asp is specific for plant DEG15 and possibly DEG15 from cellular slime molds (Fig. S7). Additionally, the analysis revealed that the N-terminal CaM-binding site is highly conserved among DEG15 homologues of higher plants, but is not present in peroxisomal processing proteases from moss, animals and cellular slime molds, suggesting that this regulatory domain is both specific to as well as conserved in higher plants (monocots and dicots). A comparison of plant DEG15 specific domains further revealed a conserved GPDP linker downstream of the CaM-binding domain—possibly mediating flexibility—followed by the peptide MRKHAF or variations thereof. There is a certain difference in length, even within different DEG15 gene copies of the same plant, and the loop within the protease domain seems to be especially variable (Fig. S7). As mentioned above, the moss sequence (PhpaDEG15) shares some of these features, such as the GPDP linker and the subsequent conserved peptide. Nevertheless, a clear indication for the presence of a CaM-binding motif is missing (Fig. S7). AtDEG15 belongs to a large family of DEG proteases of which the *Arabidopsis* genome has at least 16 (Schuhmann and Adamska 2012; Schuhmann et al. 2012). Of all AtDEG proteins, only AtDEG15 contains a CaM-binding domain (Fig. S7). AtDEG15 is also the only AtDEG protein without a PDZ domain and the only one localized in peroxisomes (Schuhmann et al. 2011, 2012; Schuhmann and Adamska 2012), strongly indicating that it represents a special form of DEG protease and that CaM-regulation of DEG15 is not only a plant but also a peroxisome-specific trait. A phylogenetic tree (Fig. 7) analysis including all AtDEG proteases in addition to DEG15 homologues from plants, animals and cellular slime molds illustrates the unique position of DEG15. All DEG15/GPP-like plant peroxisomal/glyoxysomal processing proteases branch together in close proximity to but clearly separated from a branch containing the DEG15-like proteases from non-plant organisms. All other AtDEG proteases are localized on branches separated from DEG15 proteins.

The significance of PTS2 processing is still unknown because the catalytic properties of processed and unprocessed enzymes are similar as reported for gMDH (Gietl et al. 1996; Cox et al. 2005) and PTS2 is not processed in lower eucaryotes such as yeasts. Similarily, *Arabidopsis deg15* and *cml3* null mutants do not display growth or germination defects beyond the resistance to the inhibitory effects of IBA and 2,4-DB (Hu et al. 2012; Lingard and Bartel 2009; Schuhmann et al. 2008), Therefore, the physiological benefit to regulated removal of the PTS2 sequence and its suggested influence on certain aspects of plant peroxisome metabolism as evidenced by the evolutionary conservation of this type of regulation in plant peroxisome biogenesis, remains an even more intriguing question.

## Electronic supplementary material

Below is the link to the electronic supplementary material.

Supplementary material 1 (PDF 5206 kb)

## Abbreviations

PTS: Peroxisomal targeting signal
pre-gMDH: Precursor of glyoxysomal malate dehydrogenase (gMDH)
pre-gCS: Precursor of glyoxysomal citrate synthase (gCS)
2,4-D: 2,4-Dichlorphenoxyacetic acid
2,4-DB: 2,4-Dichlorphenoxybutyric acid
IAA: Indole-3-acetic acid
IBA: Indole-3-butyric acid
IPTG: Isopropyl-β-D-thiogalactopyranosid
Y2H: Yeast two hybrid
Y3H: Yeast three hybrid
