# Supplementary material for: Calmodulin-like protein AtCML3 mediates dimerization of peroxisomal processing protease AtDEG15 and contributes to normal peroxisome metabolism
Source: Plant Mol Biol. 2013 Aug 14;83(6):607–24. doi: 10.1007/s11103-013-0112-6 (PMC3830196; doi:10.1007/s11103-013-0112-6)
Supplement: Supplementary file 1 — Supplementary material 1 (PDF 5206 kb) [file 11103_2013_112_MOESM1_ESM.pdf]

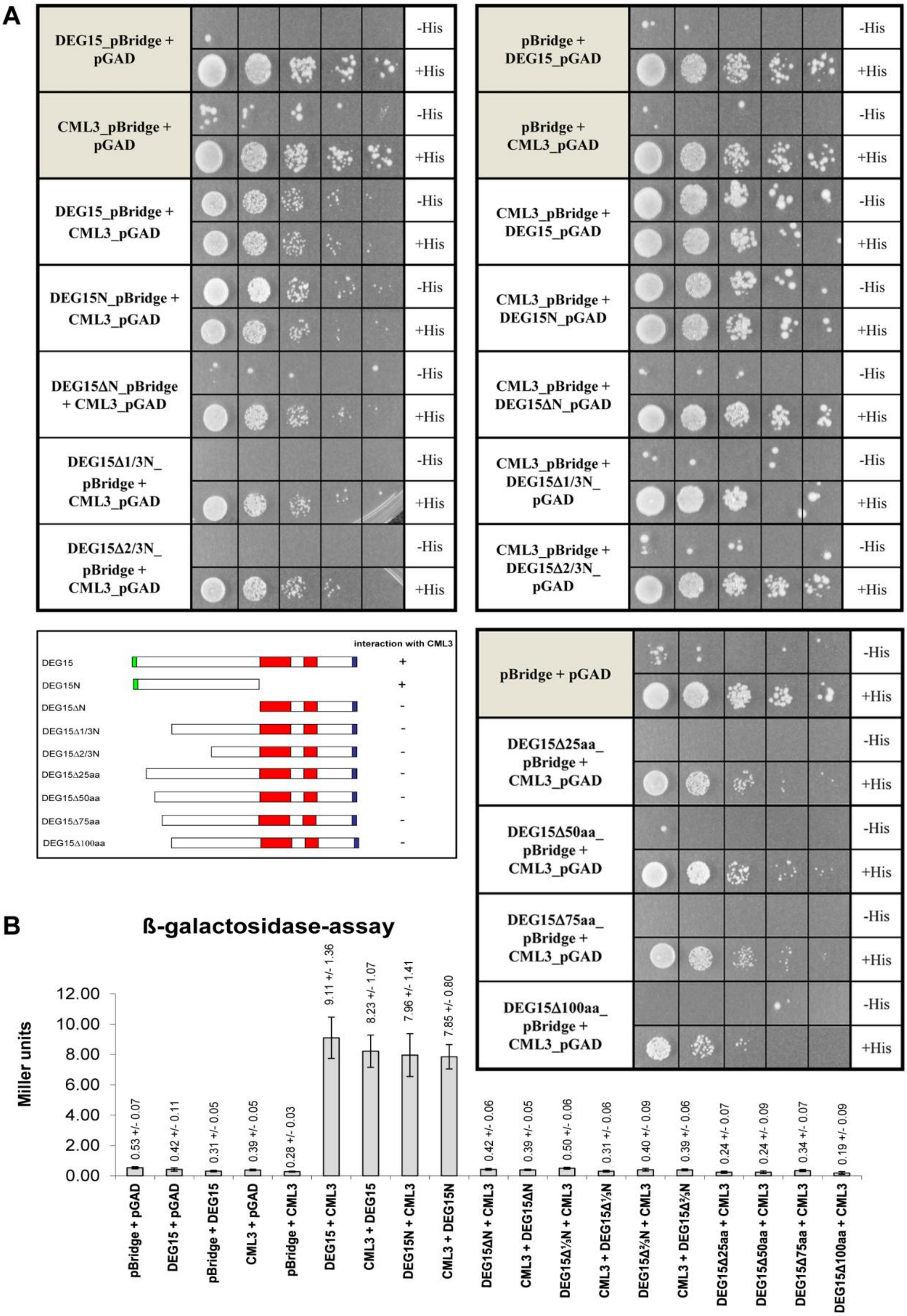

A
DEG15_pBridge + pGAD
CML3_pBridge + pGAD
DEG15_pBridge + CML3_pGAD
DEG15N_pBridge + CML3_pGAD
DEG15ΔN_pBridge + CML3_pGAD
DEG15Δ1/3N_ pBridge + CML3_pGAD
DEG15Δ2/3N_ pBridge + CML3_pGAD
pBridge + DEG15_pGAD
pBridge + CML3_pGAD
CML3_pBridge + DEG15_pGAD
CML3_pBridge + DEG15N_pGAD
CML3_pBridge + DEG15ΔN_pGAD
CML3_pBridge + DEG15Δ1/3N_ pGAD
CML3_pBridge + DEG15Δ2/3N_ pGAD
pBridge + pGAD
DEG15Δ25aa_ pBridge + CML3_pGAD
DEG15Δ50aa_ pBridge + CML3_pGAD
DEG15Δ75aa_ pBridge + CML3_pGAD
DEG15Δ100aa_ pBridge + CML3_pGAD
-His
+His
interaction with CML3
DEG15 +
DEG15N +
DEG15ΔN -
DEG15Δ1/3N -
DEG15Δ2/3N -
DEG15Δ25aa -
DEG15Δ50aa -
DEG15Δ75aa -
DEG15Δ100aa -
B
ß-galactosidase-assay
Miller units
12.00
10.00
8.00
6.00
4.00
2.00
0.00
0.53 +/- 0.07
0.42 +/- 0.11
0.31 +/- 0.05
0.39 +/- 0.05
0.28 +/- 0.03
9.11 +/- 1.36
8.23 +/- 1.07
7.96 +/- 1.41
7.85 +/- 0.80
0.42 +/- 0.06
0.39 +/- 0.05
0.50 +/- 0.06
0.31 +/- 0.06
0.40 +/- 0.09
0.39 +/- 0.06
0.24 +/- 0.07
0.24 +/- 0.09
0.34 +/- 0.07
0.19 +/- 0.09
pBridge + pGAD
DEG15 + pGAD
pBridge + DEG15
CML3 + pGAD
pBridge + CML3
DEG15 + CML3
CML3 + DEG15
DEG15N + CML3
CML3 + DEG15N
DEG15ΔN + CML3
CML3 + DEG15ΔN
DEG15Δ⅓N + CML3
CML3 + DEG15Δ⅓N
DEG15Δ⅔N + CML3
CML3 + DEG15Δ⅔N
DEG15Δ25aa + CML3
DEG15Δ50aa + CML3
DEG15Δ75aa + CML3
DEG15Δ100aa + CML3

Figure S1. AtDEG15 interacts with AtCML3 through its N-terminal 25 amino acids. Interaction assay using Y2H was tested by cloning the full-length coding regions of *AtDEG15* and its truncated variants and also *AtCML3* into pBridge and pGAD424, fusing them with the DNA-binding domain or with the DNA-activation domain, respectively, and tested for interaction in both combinations. As negative controls a combination of empty vectors and vectors harbouring one of the constructs were used. (A) Growth on His-free medium (-His) indicates interaction by transcription of the His3-reporter; (+His), positive growth control. Negative controls are shaded in grey. Yeast cultures were diluted from 10^5 cells (left) to 10^1 cells (right) per 50 µl drop applied. (B) Interaction is also demonstrated by transcription of the lacZ reporter (ß-gal activity in Miller units). Mean (n=3) +/- SD. Inlay: Schematic representation of AtDEG15 and the various deletion constructs used in A and B: green, AtCML3 binding domain; red, protease domain with plant-specific loop; blue, PTS1.

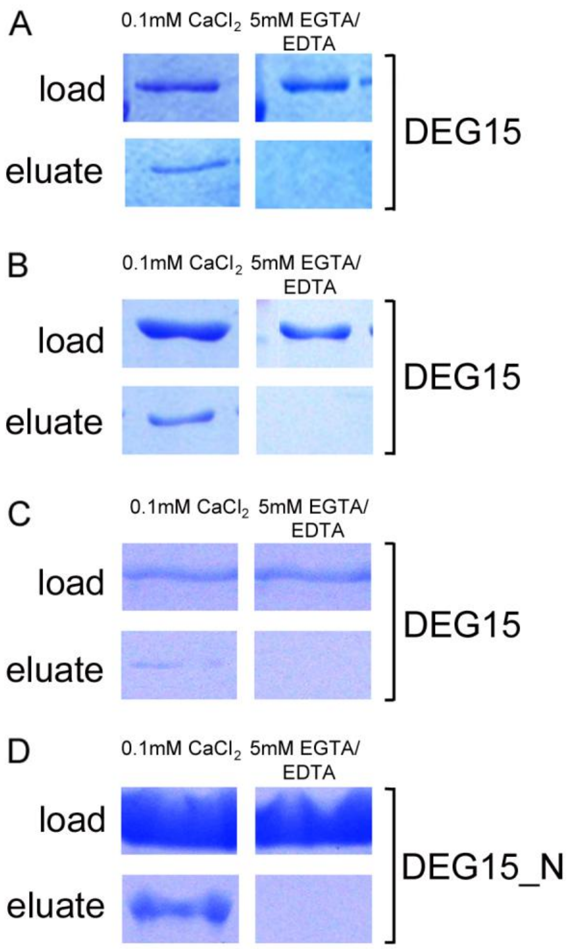

Figure S2. Binding of AtDEG15 to CaM via its N-terminal domain was confirmed by affinity chromatography on CaM-agarose. (A and B): AtDEG15 was purified from inclusion bodies using 4 M urea and incubated with CaM-agarose in the presence (0.1 mM $CaCl_2$) or absence of Ca^{2+} (5mM EGTA/EDTA). Bound protein was eluted with either bovine CaM (A) or purified recombinant AtCML3 (B). (C): Affinity chromatography on CaM-agarose using AtDEG15 purified under native conditions. AtDEG15 bound specifically to the matrix in the presence but not in the absence of Ca^{2+}, and could be eluted with bovine CaM. (D): DEG15_N comprising only the first 327 amino acids without the protease and C-terminal domains is still able to bind to calmodulin-agarose in a calcium-dependent manner.

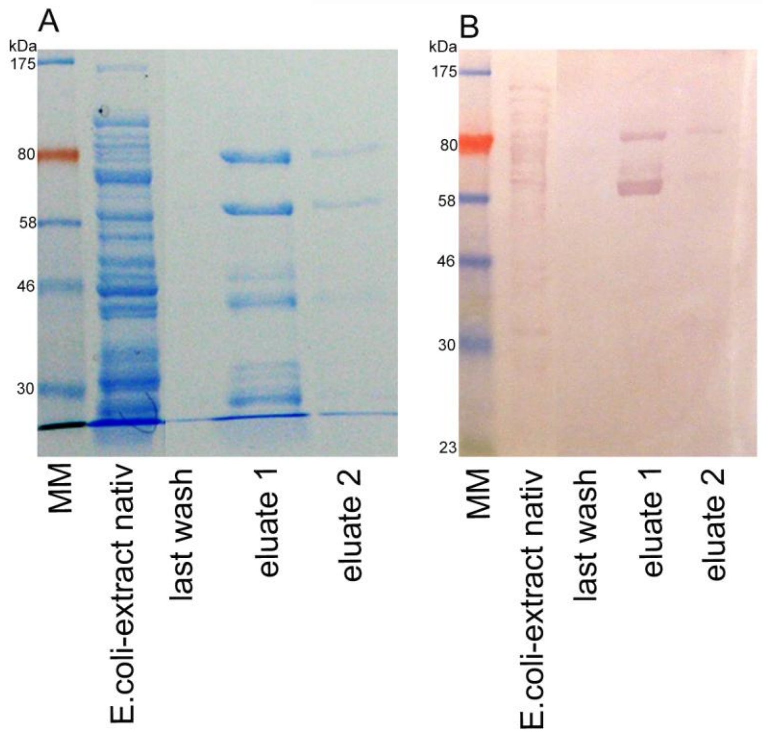

Figure S3. Recombinant expression of soluble AtDEG15 yields the full length protein consistently accompanied by various degradation products. AtDEG15 with an N-terminal His-tag and an N-terminal "Xpress epitope" permitting detection of the recombinant fusion protein by appropriate antibodies was expressed in *E.coli* under control of an arabinose-inducible promoter. The protein was purified by HisTrap affinity chromatography under native conditions. Analysis of all fractions by SDS-PAGE and Coomassie staining (A) or Westernblot (B) revealed the full length AtDEG15 protein (76 kDa) as well as varying degradation products probably due to self cleavage.

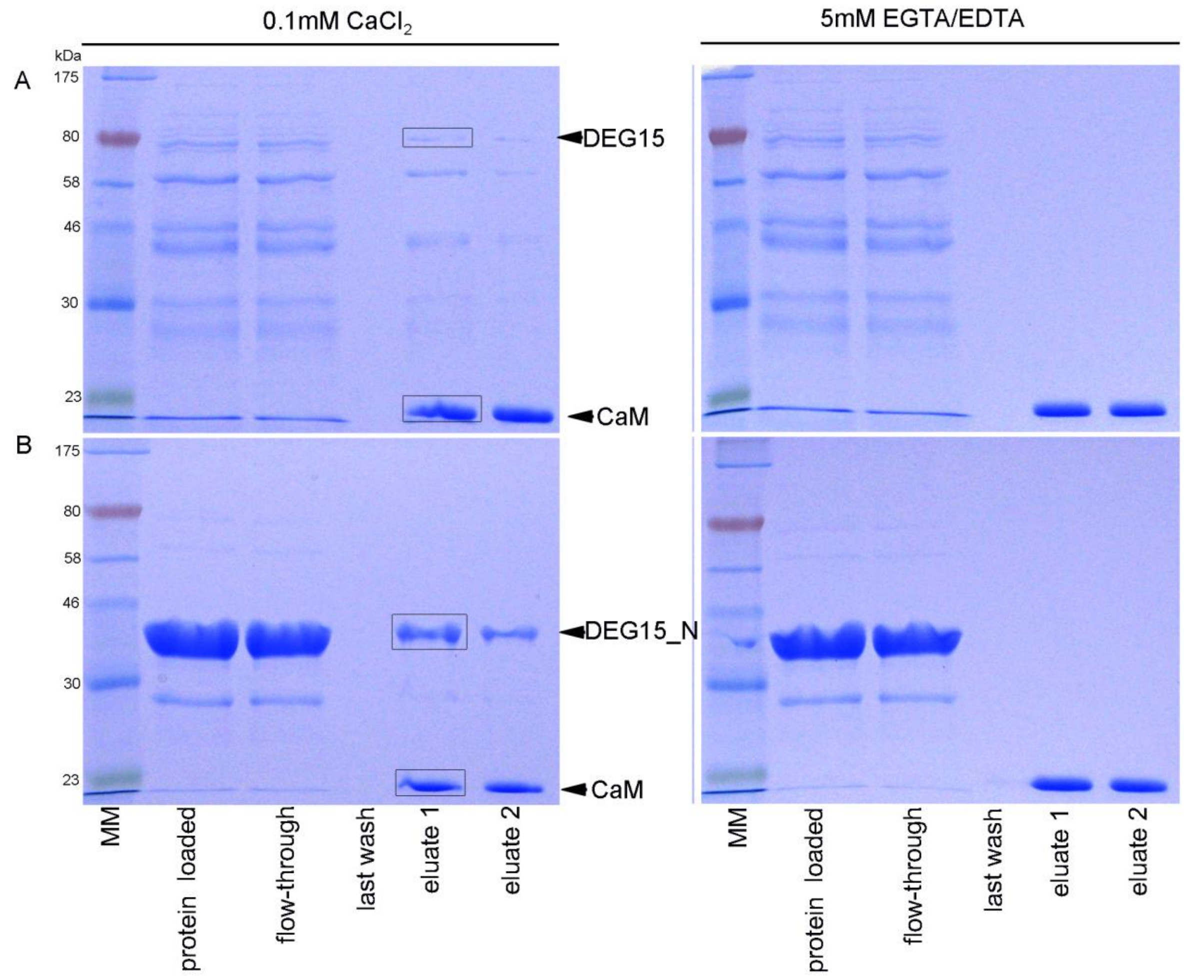

Figure S4. Recombinant enzymatically active full length AtDEG15 exhibits the characterics of the monomer as a general protease with an intrinsic self-cleavage activity. (A) His-tagged purified AtDEG15 comprising full length protein and C-terminally truncated degradation products was incubated over night at 4°C before affinity chromatography on CaM-agarose under native conditions in the presence (0.1 mM $CaCl_2$) and absence of Ca^{2+} (5mM EGTA/EDTA). Purified AtDEG15 incubated overnight as well as the different fractions of the affinity chromatography on CaM-agarose were analyzed by SDS-PAGE and Coomassie staining. AtDEG15 full length protein together with its C-terminally truncated versions bound specifically to the matrix in the presence of Ca^{2+} and could be eluted with bovine CaM, but not in the absence of Ca^{2+} (5mM EGTA/EDTA), whereas N-terminally truncated versions could not bind to the CaM-ligand. (B) The AtDEG15_N-terminus lacking the protease domain was stable under these experimental conditions and also bound to the CaM-agarose in a calcium-dependent fashion..

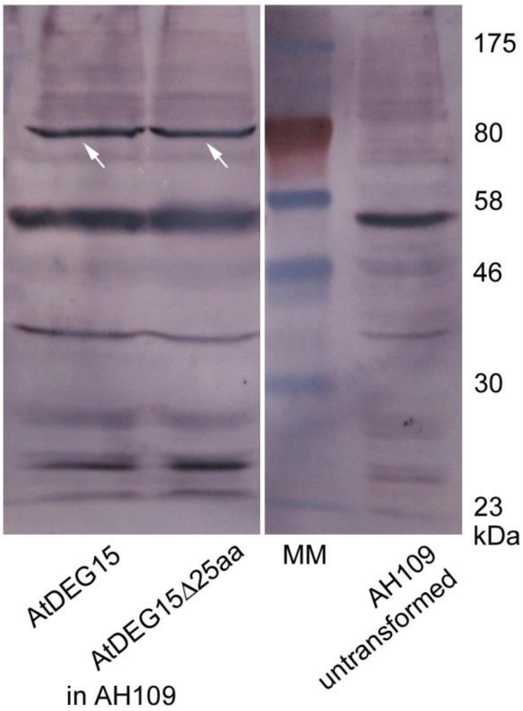

Figure S5. Western Blot analysis of yeast AH109 cells expressing the full-length AtDeg15 and the AtDeg15Δ25aa variant with antibodies directed against the binding domain in pBRIDGE [GAL4 (DBD) (RK5C1) Santa Cruz Biotechnology] show immuno-reactive bands of the expected size in both strains and not in the untransformed yeast AH109 used as a negative control.

A

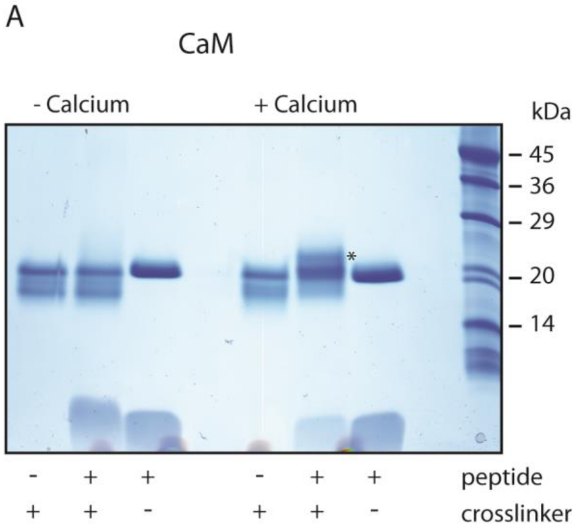

B

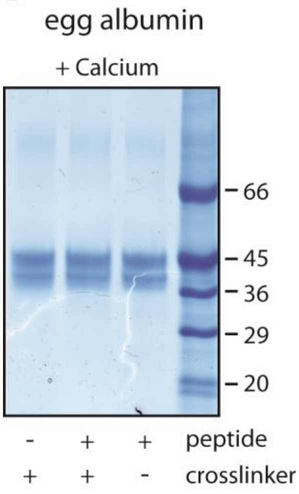

Figure S6. Crosslinking of a synthetic AtDEG15 peptide with CaM. Crosslinking experiments were performed using a synthetic peptide comprising the first 21 amino acids of AtDEG15 (H2N - MDVSKVVSFSRNFAVLVKVEG - CONH2) and either CaM (A) or egg ovalbumin (B). A cross-linking product between CaM and the peptide can be observed solely in the presence of calcium. No cross-linking product occurs when CaM is replaced by egg albumin.

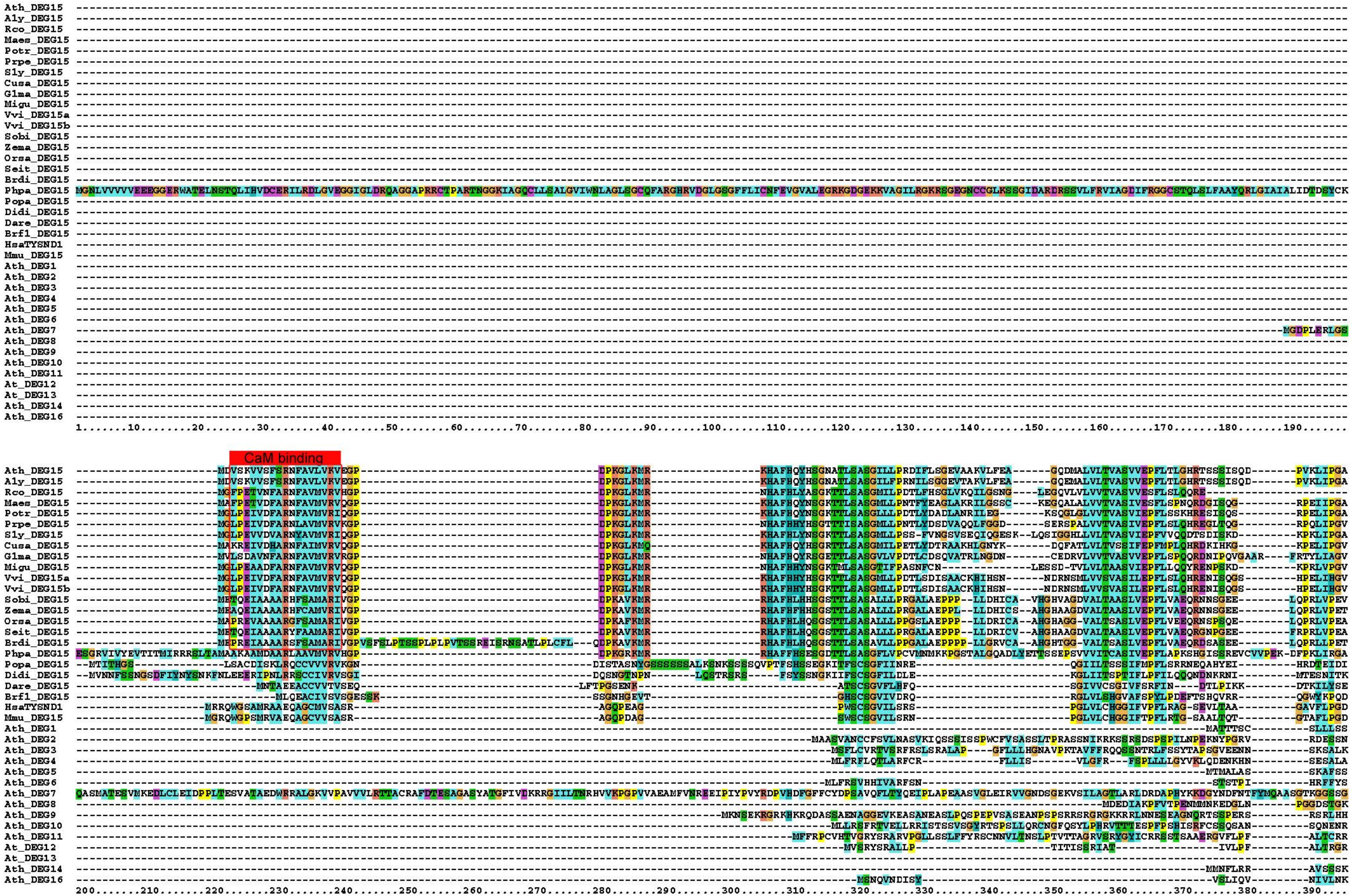

CaM binding
Ath_DEG15
Aly_DEG15
Rco_DEG15
Maes_DEG15
Potr_DEG15
Prpe_DEG15
Sly_DEG15
Cusa_DEG15
Glma_DEG15
Migu_DEG15
Vvi_DEG15a
Vvi_DEG15b
Sobi_DEG15
Zema_DEG15
Orsa_DEG15
Seit_DEG15
Brdi_DEG15
Phpa_DEG15
Popa_DEG15
Didi_DEG15
Dare_DEG15
Brfl_DEG15
HsaTYSND1
Mmu_DEG15
Ath_DEG1
Ath_DEG2
Ath_DEG3
Ath_DEG4
Ath_DEG5
Ath_DEG6
Ath_DEG7
Ath_DEG8
Ath_DEG9
Ath_DEG10
Ath_DEG11
At_DEG12
At_DEG13
Ath_DEG14
Ath_DEG16

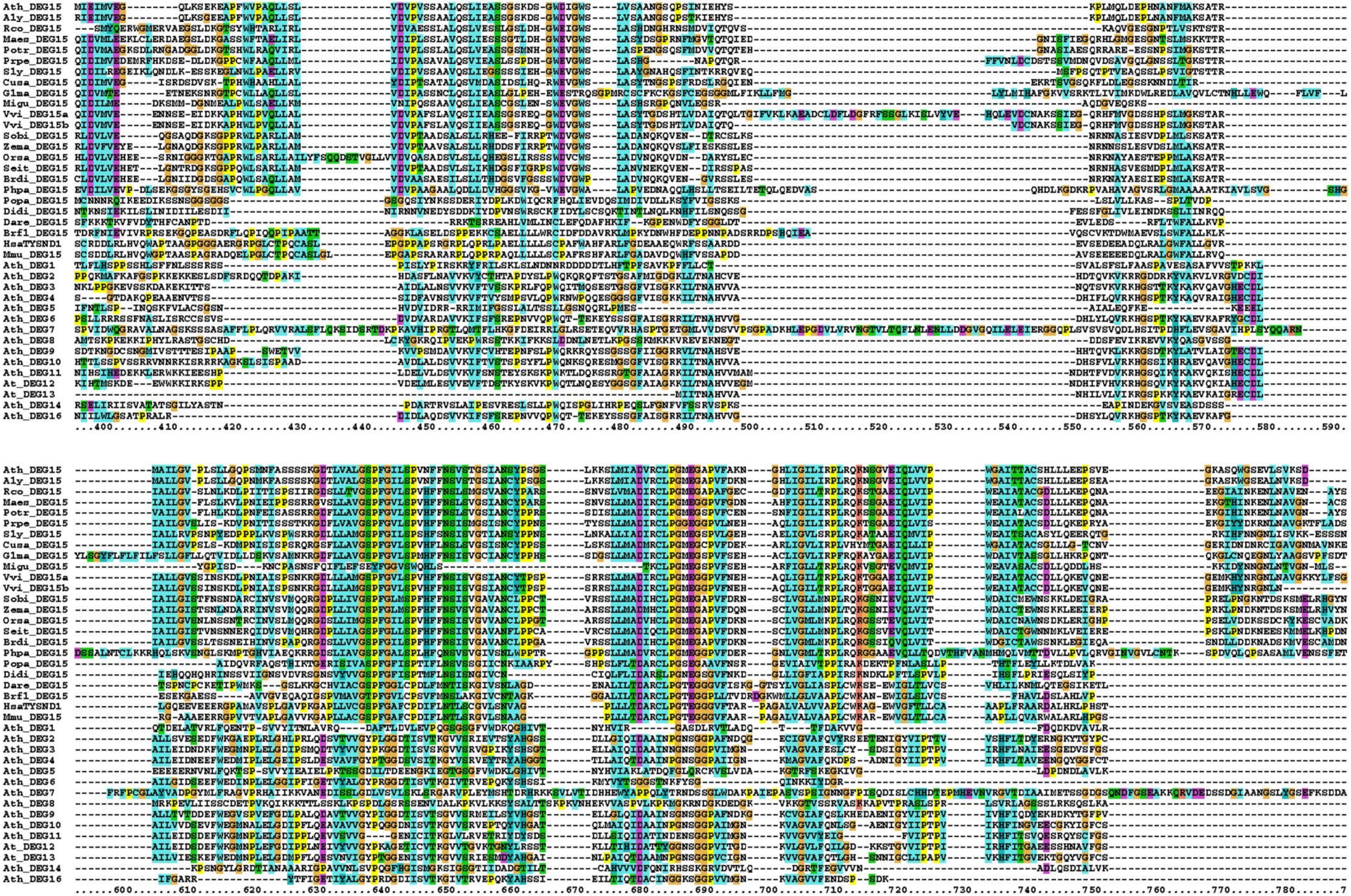
Ath_DEG15
Aly_DEG15
Rco_DEG15
Maes_DEG15
Potr_DEG15
Prpe_DEG15
Sly_DEG15
Cusa_DEG15
Glma_DEG15
Migu_DEG15
Vvi_DEG15a
Vvi_DEG15b
Sobi_DEG15
Zema_DEG15
Orsa_DEG15
Seit_DEG15
Brdi_DEG15
Phpa_DEG15
Popa_DEG15
Didi_DEG15
Dare_DEG15
Brfl_DEG15
HsaTYSND1
Mmu_DEG15
Ath_DEG1
Ath_DEG2
Ath_DEG3
Ath_DEG4
Ath_DEG5
Ath_DEG6
Ath_DEG7
Ath_DEG8
Ath_DEG9
Ath_DEG10
Ath_DEG11
At_DEG12
At_DEG13
Ath_DEG14
Ath_DEG16

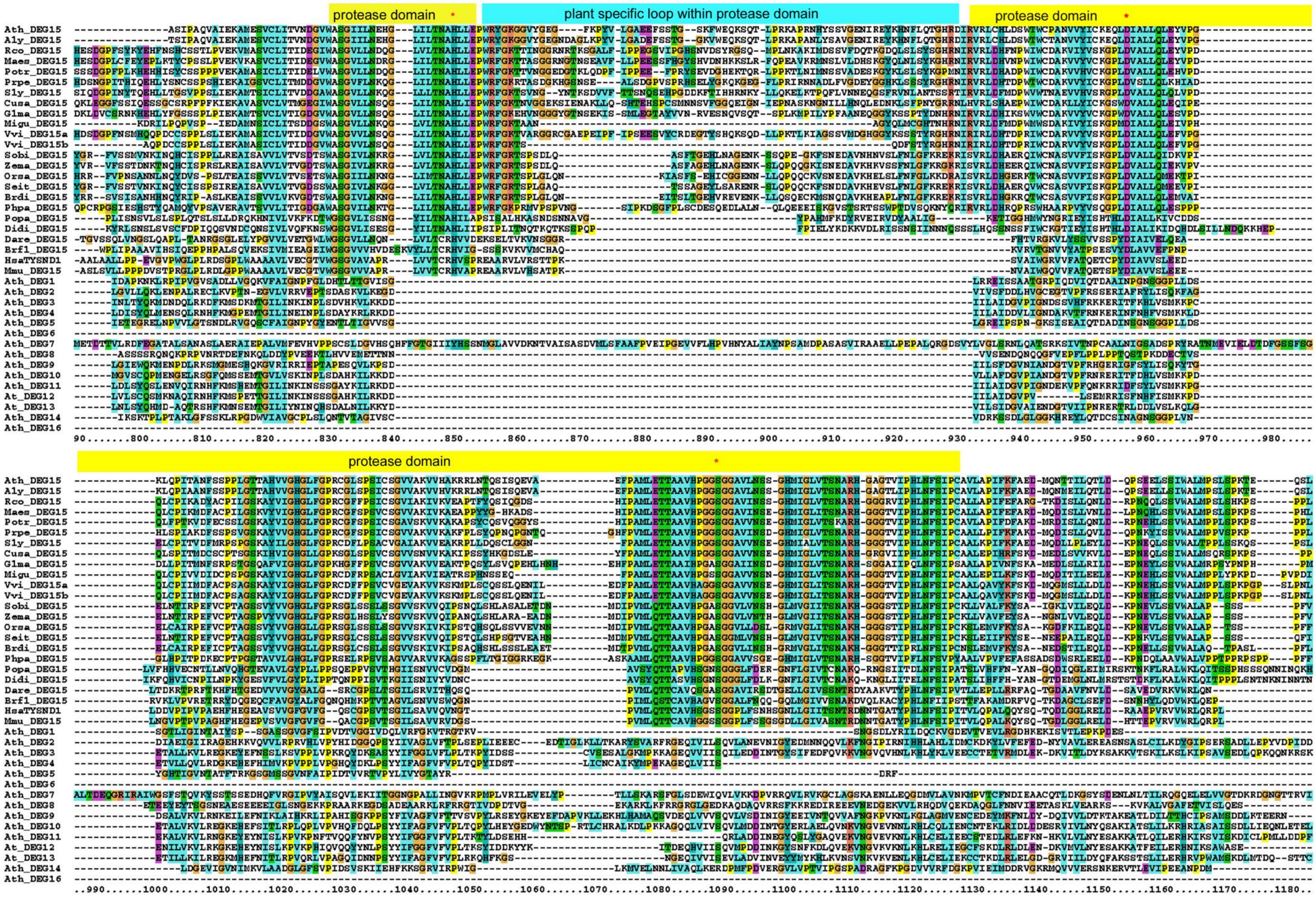
protease domain
plant specific loop within protease domain
protease domain
protease domain

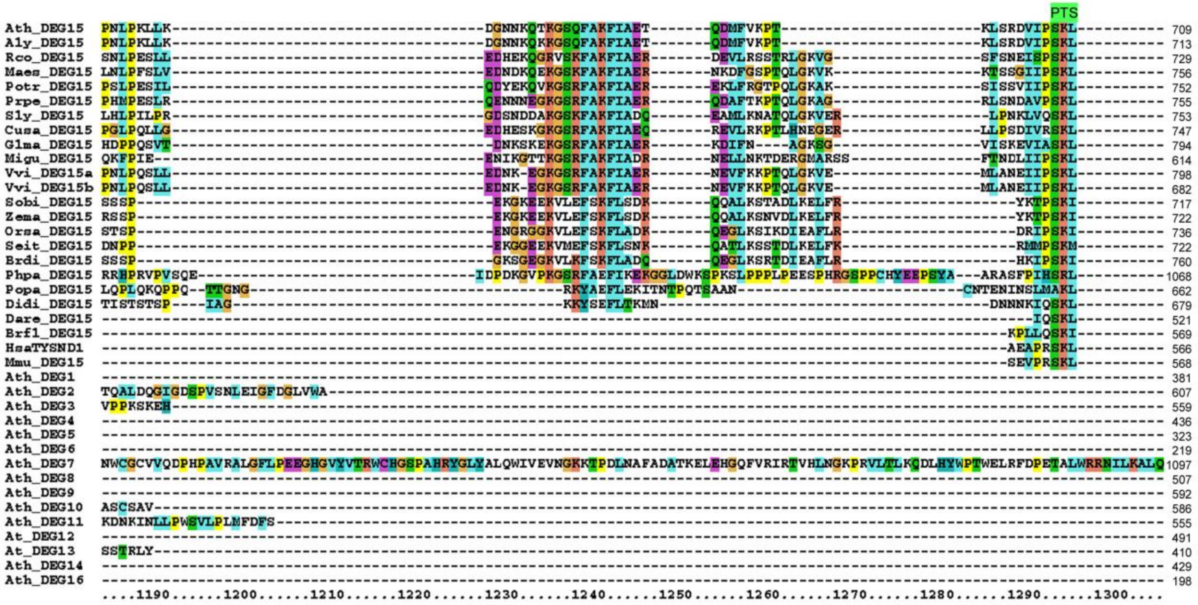

Figure S7. Sequence alignement of peroxisomal processing proteases from plants, animals and cellular slime molds and of DEG proteases in Arabidopsis used for construction of the phylogenetic tree (Fig. 7). The CaM-binding domain at the N-terminus of DEG15 from dicots and monocots (also boxed in red within the sequences), the protease domain in the plant, animal and cellular slime mold DEG15 with their catalytic triade His, Asp and Ser (*), the plant specific loop within the protease domain and the PTS1 at the C-terminus are indicated above the sequences. Amino acids conserved among the sequences are coloured according to the *Clustal X Colour Scheme*: orange, G; yellow, P; green, T, S, N, Q; blue, W, L, V, I, M, A, F; cyan: H, Y; magenta: E, D; red: K, R. For accession numbers see supplemental Tab. S2A. Sequence alignments were obtained by ClustalX 2.0 [Thompson JD, Gibson TJ, Plewniak F, Jeanmougin F, Higgins DG (1997) The CLUSTAL_X windows interface: flexible strategies for multiple sequence alignment aided by quality analysis tools. Nucleic Acids Res 25:4876-4882] and phylogenetic tree construction was performed by maximum likelihood using tree-puzzle-5.2 [Strimmer K, von Haeseler A (1996) Quartet puzzling: A quartet maximum-likelihood method for reconstructing tree topologies. Mol Biol Evol 13:964-969].

Figure S8: Amino acids of the entire beta-casein sequence (224 aa) including the pre-sequence (amino acid residues 1-15) and the mature beta-casein (amino acid residues 16-224) with the AtDEG15 cleavage sites indicated by arrows.

```
MKVLILACLVALALA-
RELEELNVPGEIVESLSSSEESITRINKKIEKFQSEEQQQTEDELQDKIHPFAQTQSLVYPFPGPIPNSLPQNIP
PLTQTPVVVPPFLQPEVMGVSKVKEAMAPKHKEMPFPKYPVEPFTESQSLTLT↓DVENLHLPLPLLQS↓W↓M↓HQ
PHQPLPPTVM↓FPPQ↓S↓VLSLSQSKVLPVPQKAVPYPQRDMPIQAFLL↓YQEPV↓LGPVR↓GPFPIIV
```

Beta-casein peptides obtained by cleavage with recombinant AtDEG15

aa-position beta-casein	peptide sequence
144-157	T‖DVENLHLPLPLLQS‖W
159-171	W‖MHQPHQPLPPTVM‖F
159-176	W‖MHQPHQPLPPTVMFPPQS‖V
160-171	M‖HQPHQPLPPTVM‖F
160-175	M‖HQPHQPLPPTVMFPPQ‖S
160-176	M‖HQPHQPLPPTVMFPPQS‖V
208-217	L‖YQEPVLGPVR‖G
213-224 C-term	V‖LGPVRGPFPIIV.-

Figure S9. *Dictyostelium discoideum* DEG15. XP_641938: The protein sequence as it is published in the database contains long repeat regions of N, Q and S that don't match the other cellular slime mold *Polysphondylium pallidum* DEG15 and would cause long gaps in the alignment of DEG proteins (Fig. S4). DiDi_DEG15: Protein sequence without the long repeat regions of N, Q and S as it was used for the alignment of DEG proteins.

```
>XP_641938
MVNNFSSNGSDFINNINNNNNNNSNNNSNNNNYNYSNKFNLEEERIPNLRRSCCIVRVSGIDQSNGTNPNLQSTR
SRSFSYSSNGKIIFSCSGFILDLEKGLIITSPTIFLPFILQQQNDNKRNINNNNNNNNNNNNNNQMTESNITKNT
KNSIEKILSLINIDIILESDIINIRNNVNENNNNNDYSDDKIYDPVNSWRSCKFIDYLSCSQKTINTLNQLKNHF
ILSNQSSGFESSFGLIVLEINDKSSLIINQQQQQQRQQIEHQQHQHQQQQQRINSSVIIGNSVDVRSGNSVYVVG
SPFGFISPTMFLNSISNGIVCNCIQSSSSSSSSLFLIDARSLPGNEGSGVFNKDGLLIGFIAPPIRSKNDKLPFT
LSPVLPIHSFLPRIESQLSIYPKYRLSNSLSVSCFDPIQQSVNDCQNSIVLVQFKNSWGSGVLISESGYILTNAH
LIIPSIPLIQQQIQQQQQQQQQQQQQQQQQQQQQQTNQTKQTKSSQQQQQQQQQQQQPQPFPIELYKDKKVDLRI
SSNSIINNNQSSSLHQSSNSSFIWCKGTIEYISHTHLDIALIKIDQHDLSILLNDNNNNNNNNNNNNNNNNNNNN
NNNNNNNNNNNNNNNNNNNNNNNNNNQKKHEPIKFQHVICNPILNPKYGESVFVLGYPLIPPTQNPPISVTKGII
SNIVYVDNCAVSYQTTASVHSGNSGGGLFDLKGNFLGIVTCNAKQKNGLIITELNFSIPATSLIHFFHYANGTDE
MGLNLMRSTSTDKFLKALWKLQITPPPLSNTNKNINNTNTNNNNNNNNNNNNNNNNNNNNNNNISTSTSPIAGKK
YSEFLTKMNQQQQQDNNNKIQSKL

>DiDi_DEG15
MVNNFSSNGSDFIYNYSNKFNLEEERIPNLRRSCCIVRVSGIDQSNGTNPNLQSTRSRSFSYSSNGKIIFSCSGF
ILDLEKGLIITSPTIFLPFILQQQNDNKRNIMTESNITKNTKNSIEKILSLINIDIILESDIINIRNNVNEDYSD
DKIYDPVNSWRSCKFIDYLSCSQKTINTLNQLKNHFILSNQSSGFESSFGLIVLEINDKSSLIINRQQIEHQQHQ
HRINSSVIIGNSVDVRSGNSVYVVGSPFGFISPTMFLNSISNGIVCNCIQLFLIDARSLPGNEGSGVFNKDGLLI
GFIAPPIRSKNDKLPFTLSPVLPIHSFLPRIESQLSIYPKYRLSNSLSVSCFDPIQQSVNDCQNSIVLVQFKNSW
GSGVLISESGYILTNAHLIIPSIPLITNQTKQTKSSPQPFPIELYKDKKVDLRISSNSIINNNQSSSLHQSSNSS
FIWCKGTIEYISHTHLDIALIKIDQHDLSILLNDQKKHEPIKFQHVICNPILNPKYGESVFVLGYPLIPPTQNPP
ISVTKGIISNIVYVDNCAVSYQTTASVHSGNSGGGLFDLKGNFLGIVTCNAKQKNGLIITELNFSIPATSLIHFF
HYANGTDEMGLNLMRSTSTDKFLKALWKLQITPPPLSNTNKNINNTNTISTSTSPIAGKKYSEFLTKMNDNNNKI
QSKL
```

Table S1. List of primers used in this study

Primer name	5´-3´sequence
Y2HDEG15fw	ACGTACGAATTCATGGATGTGTCTAAAG
Y2HDEG15rev	TGCATCGTCGACAGGGATCACATC
Y2HDEG15Δ1/3N fw	TCGATCGAATTCAGTAGTAGTATCTCTC
Y2HDEG15Δ2/3N fw	TCGATCGAATTCGTCCCCTTAAGTTTAC
Y2HDEG15ΔN fw	TCGATCGAATTCGCACAAGTGGCTATTG
Y2HDEG15N rev	AGCTAGGTCGACAACACCATCATTG
Y2HDEG15ΔC rev	AGCTAGGTCGACTCAGGTGCCAAGACTGC
Y2HDEG15Δ25AS fw	GCATCGAATTCATGTTGAAGATGAGGAAGCATGC
Y2HDEG15Δ50AS fw	GCATCGAATTCATGCCAAGAGATATTTTTTTGAG
Y2HDEG15Δ75AS fw	GCATCGAATTCATGTTGACCGTTGCATCTGTTG
Y2HDEG15Δ100AS fw	GCATCGAATTCATGGTGAAGCTTATTCCGGGTGC
Y2HCAM fw	ACGTACGAATTCATGGATCAAGCGGAGC
Y2HCAM rev	TGCATGGTCGACTCCTAAGGCGGC
Y3HCAM fw	AAGGTGGCGGCCGCATGGATCAAGCGGAGCTTGC
Y3HCAM rev	CCCGAAGATCTTTACAAGTTTGATCCTAAGGCG
pETDEG15fw	CGATCGATCGAATTCTGGATGTGTCTAAAGTTGTCAGC
pETDEG15rev	CCTTAGCTCGAGTCATAACTTGCTAGGGATCACATCACG
pETDEG15Δ1/3N fw	CGATCGATCGAATTCAGTAGTAGTATCTCTCAGGATCCGG
pETDEG15Δ2/3N fw	CGATCGATCGAATTCGTCCCCTTAAGTTTACTGGGTCAACC
pETDEG15ΔN fw	CGATCGATCGAATTCTGGGCTTCTGGTATTATTCTTAACG
pETDEG15Δloop fw	CACCTCAGGAGCTCCTCAAGCAGGTGAGCATTTG
pETDEG15Δloop rev	CGATCGATCGAGCTCGGGCATAGAGACATACGTGTGCG
pETDEG15ΔC rev	CTCAGCTCGAGTCATAACTTGCTACATGGGATGCTGAAGTTGAGATGCGG
pETCML3 fw	ATCGATCGAATTCATGGATCAAGCGGAGCTTGC
pETCML3 rev	CCTTAGGTCGACCAAGTTTGATCCTAAGG
pCAMBIA fw	CGATCGATCAGATCTATGGGCAGCAGCCATCATCATC
pCAMBIA ΔC rev	CCTCAGCACGTGCTCGAGTCATAACTTGCTAGGGATC
pCAMBIA rev	CCTCAGCACGTGCTCGAGTCATAACTTGCTACATGG
pCAMBIA CML3-bd fw	ACTGACCATGGACATGGATGTGTCTAAAG
pCAMBIA CML3-bd rev	TCAGTAGATCTAACTTTGGATCAGGGCC

fw, forward; rev, reverse.

Table S2A: DEG proteases from plants, animals and cellular slime molds for alignement (Fig. S7) and phylogenetic tree (Fig. 7)

Denotation	Organism	Accession-Nr.	Localization	Domain-structure
Ath_DEG15	*Arabidopsis thaliana*	NP_174153	SKL (peroxisome)	no PDZ-domain
Aly_DEG15	*Arabidopsis lyrata*	XP_002893523	SKL (peroxisome)	no PDZ-domain
Rco_DEG15	*Ricinus communis*	XP_002509448	SKL (peroxisome)	no PDZ-domain
Potr_DEG15	*Populus trichocarpa*	XP_002305124/POPTR_4s04650	SKL (peroxisome)	no PDZ-domain
Cusa_DEG15	*Cucumis sativus*	Cucsa046320	SKL (peroxisome)	no PDZ-domain
Glma_DEG15	*Glycine max*	Glyma13g31080	SKL (peroxisome)	no PDZ-domain
Migu_DEG15	*Mimulus guttatus*	mgf007197	SKL (peroxisome)	no PDZ-domain
Prpe_DEG15	*Prunus persica*	ppa001854	SKL (peroxisome)	no PDZ-domain
Maes_DEG15	*Manihot esculenta*	4.1_003779m + 4.1_018442m.g	SKL (peroxisome)	no PDZ-domain
Vvia_DEG15	*Vitis vinifera*	XP_002273997	SKL (peroxisome)	no PDZ-domain
Vvib_DEG15	*Vitis vinifera*	CBI30593	SKL (peroxisome)	no PDZ-domain
Sobi_DEG15	*Sorghum bicolor*	XP_002441322	SKI (peroxisome)	no PDZ-domain
Zema_DEG15	*Zea mays*	GRMZM2G162699	SKI (peroxisome)	no PDZ-domain
Orsa_DEG15	*Oryza sativa*	EEE64231	SKI (peroxisome)	no PDZ-domain
Seit_DEG15	*Setaria italica*	SiPROV003192	SKM (peroxisome)	no PDZ-domain
Brdi_DEG15	*Brachypodium distachyon*	Bradi2g21020	SKI (peroxisome)	no PDZ-domain
Phpa_DEG15	*Physcomitrella patens*	XP_001775762	SRL (peroxisome)	no PDZ-domain
Popa_DEG15	*Polysphondylium pallidum*	EFA82668	AKL (peroxisome)	no PDZ-domain
Didi_DEG15	*Dictyostelium discoideum*	XP_641938	SKL (peroxisome)	no PDZ-domain
Darer_NP_001	*Danio rerio*	XP_690168	SKL (peroxisome)	no PDZ-domain
Brfl_DEG15	*Branchyostome floridae*	XP_002609926	SKI (peroxisome)	no PDZ-domain
HsaTYSND1	*Homo sapiens*	NM_173555	SKL (peroxisome)	no PDZ-domain
Mumu_NP082188	*Mus musculus*	XP_125636 XP_002509448	SKL (peroxisome)	no PDZ-domain
Ath_DEG1	*Arabidopsis thaliana*	XM_002667490	chloroplasts	1 PDZ-domain
Ath_DEG2	*Arabidopsis thaliana*	AF245171	chloroplasts	1 PDZ-domain, elongated C-terminus
Ath_DEG3	*Arabidopsis thaliana*	NM_105236	mitochondria	1 PDZ-domain, elongated C-terminus
Ath_DEG4	*Arabidopsis thaliana*	NM_105237	mitochondria	1 PDZ-domain, elongated C-terminus
Ath_DEG5	*Arabidopsis thaliana*	NM_117947	chloroplasts	truncated, no PDZ-domain
Ath_DEG6	*Arabidopsis thaliana*	NM_103994	chloroplasts	no PDZ-domain
Ath_DEG7	*Arabidopsis thaliana*	NM_111209	nucleus (?)	2 protease-domains (1 inactiv), 4 PDZ-domains
Ath_DEG8	*Arabidopsis thaliana*	NM_123346	chloroplasts	1 PDZ-domain
Ath_DEG9	*Arabidopsis thaliana*	NM_123384	chloroplasts	1 PDZ-domain, elongated C-terminus
Ath_DEG10	*Arabidopsis thaliana*	NM_123053	mitochondria	1 PDZ-domain, elongated C-terminus
Ath_DEG11	*Arabidopsis thaliana*	NM_112526	mitochondria	1 PDZ-domain, elongated C-terminus
Ath_DEG12	*Arabidopsis thaliana*	NM_112527	mitochondria	1 PDZ-domain, elongated C-terminus
Ath_DEG13	*Arabidopsis thaliana*	NM_123420	unknown	1 PDZ-domain, elongated C-terminus
Ath_DEG14	*Arabidopsis thaliana*	NM_122648	mitochondria	1 PDZ-domain
Ath_DEG16	*Arabidopsis thaliana*	NM_148132	chloroplasts	1 PDZ-domain, elongated C-terminus

Table S2B: DEG15 proteases from plants (truncated sequences) and peroxisomal proteases from green algae

Denotation	Organism	Accession-Nr.	encoded protein	catalytic aa	PTS1
Metr_DEG15	*Medicago truncatulata*	Medtr2g017690	608 aa	His-Asp-Ser	truncated
Metr_DEG15	*Medicago truncatulata*	Medtr2g017700	194 aa	truncated	truncated
Aqco_DEG15	*Aquilegia coerulea*	AcoGoldSmith_v1.004744m.g	461 aa	His-Asp-Ser	SKL
Capa_DEG15	*Carica papaya*	evm.TU.supercontig_373.8	502 aa	His-Asp-Ser	SKL
Potr_DEG15	*Populus trichocarpa*	POPTR_0011s05510	729 aa	His-Asp-Ser	SKL
	Volvox carteri f. nagariensis	XP002956349	1088 aa	Asp-Ser	SRL
	Micromonas sp. RCC299	XP002505560	708 aa	Asp-Ser	SKL
